# InfoScan: A New Transcript Identification Tool Based on scRNA-Seq and Its Application in Glioblastoma

**DOI:** 10.3390/ijms26052208

**Published:** 2025-02-28

**Authors:** Shiqiang Mei, Jinjin Huang, Zhen Zhang, Haotian Lei, Qiaojuan Huang, Lianghu Qu, Lingling Zheng

**Affiliations:** MOE Key Laboratory of Gene Function and Regulation, State Key Laboratory for Biocontrol, Innovation Center for Evolutionary Synthetic Biology, School of Agriculture and Biotechnology, School of Life Sciences, Sun Yat-sen University, Guangzhou 510275, China; 15521312895@163.com (S.M.); huangjj259@mail2.sysu.edu.cn (J.H.); zhangzh576@mail2.sysu.edu.cn (Z.Z.); leiht@mail2.sysu.edu.cn (H.L.); huangqj@mail.sysu.edu.cn (Q.H.); lssqlh@mail.sysu.edu.cn (L.Q.)

**Keywords:** scRNA-Seq, lncRNA, GBM, neoplastic, survival, treatment

## Abstract

InfoScan is a novel bioinformatics tool designed for the comprehensive analysis of full-length single-cell RNA sequencing (scRNA-seq) data. It enables the identification of unannotated transcripts and rare cell populations, providing a powerful platform for transcriptome characterization. In this study, InfoScan was applied to glioblastoma multiforme (GBM), identifying a rare “neoplastic-stemness” subpopulation exhibiting cancer stem cell-like features. Functional analyses suggested that tumor-associated macrophages (TAMs) secrete SPP1, which binds to CD44 on neoplastic-stemness cells, activating the PI3K/AKT pathway and driving lncRNA transcription to promote metastasis. Integration of TCGA and CGGA datasets further supported these findings, highlighting key mutations associated with the neoplastic-stemness subpopulation. Drug sensitivity assays indicated that neoplastic-stemness cells might be sensitive to omipalisib, a PI3K inhibitor, pointing to a potential therapeutic target. InfoScan offers a robust framework for exploring complex transcriptomic landscapes and characterizing rare cell populations, providing valuable insights into GBM biology and advancing precision cancer therapy.

## 1. Introduction

LncRNAs are noncoding RNA molecules over 200 nucleotides long that regulate various biological processes, including gene expression [1], chromatin structure modification [2], cell cycle regulation [3], cellular differentiation [4], and apoptosis [5]. Recent studies have revealed their key roles in the pathogenesis and progression of cancers [6,7], immune responses [8,9,10], cardiovascular diseases [11,12], and neurological disorders [13]. Research indicates that 60.8% of lincRNAs show tissue-specific expression, particularly in the brain and testes, whereas only 29.4% of mRNAs do [14], suggesting that lncRNAs have more pronounced spatiotemporal and lineage-specific patterns. However, their average expression is significantly lower than that of mRNAs, at approximately 1/13.6th the average mRNA expression level [15]. Full-length scRNA-seq has emerged as a powerful tool for mapping lncRNA expression at the single-cell level, enabling the discovery of previously uncharacterized lncRNAs and novel isoforms [16,17].

Despite advances in full-length scRNA-seq technologies, tools for discovering and annotating lncRNAs and isoforms remain underdeveloped [18]. Current methods heavily rely on existing transcript datasets, limiting the identification of rare cell types and their functions. Especially, glioblastoma multiforme (GBM) is the most prevalent and aggressive primary brain tumor, which has a median survival of less than 15 months [19,20]. Therapeutic failure in GBM is linked to glioma cancer stem cells (CSCs), a rare subpopulation that drives treatment resistance and tumor relapse. However, distinguishing CSCs from other cell types remains challenging due to their low abundance and high heterogeneity [21].

Emerging studies highlight the pivotal role of lncRNAs in GBM malignancy, particularly in regulating stemness, therapy resistance, and immune evasion [22,23].For example, lncRNA HOTAIR has been reported to promote cell cycle progression and invasion in GBM via an interaction with the polycomb repressive complex 2 (PRC2) and the modulation of histone methylation [24]. Additionally, MALAT1 (metastasis-associated lung adenocarcinoma transcript 1) is implicated in glioma proliferation and migration by modulating transcription factors and splicing regulators [25]. Another extensively studied lncRNA, NEAT1 (nuclear-enriched abundant transcript 1), functions within nuclear paraspeckles and has been associated with glioma cell viability, invasion, and therapeutic responses [26]. Beyond these well-known examples, lncRNAs such as H19 and TUG1 (taurine upregulated gene 1) have also been found to promote cell growth and migration in GBM models [27,28]. H19 can serve as a competing endogenous RNA, sponging microRNAs that regulate genes involved in glioma proliferation, while TUG1 has been linked to stemness features and aggressive tumor behavior [27,28].

In this study, InfoScan, a cross-platform tool, was developed to analyze, identify, and annotate novel lncRNAs from full-length scRNA-seq data. When applied to GBM samples, InfoScan firstly revealed numerous previously unannotated lncRNAs. Then, using a NovelScore method to assess transcript activity, a rare GBM cell population exhibiting prominent neoplastic stem cell properties, termed “neoplastic-stemness”, was identified. Following this, Gene coexpression analysis and cell communication investigations suggested that in the TME, cells may release SPP1, which could bind to CD44, potentially activating the PI3K/AKT pathway and lncRNA transcription and driving neoplastic-stemness development. Finally, InfoScan further linked the neoplastic-stemness population to poor survival outcomes in two independent cohorts, hinting at its potential diagnostic and therapeutic significance for glioblastoma.

## 2. Results

### 2.1. Establishment of the InfoScan Tool

**Figure 1:**
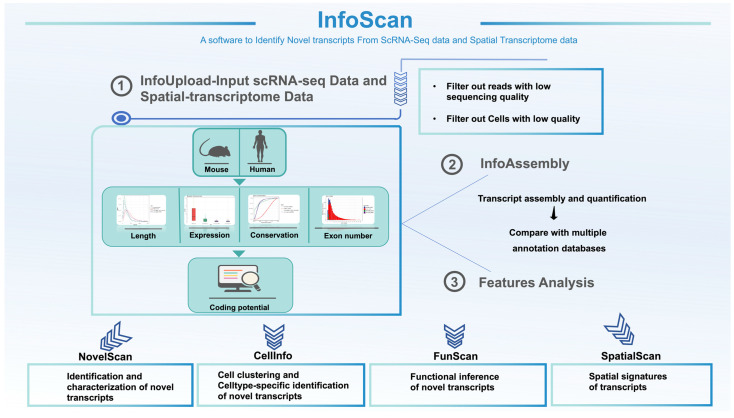
Overview of InfoScan.

InfoScan is a versatile tool designed for the analysis and identification of full-length transcripts from scRNA-seq data and features six core modules (Figure 1). Through a user-friendly interface, the InfoUpload module enables scRNA-seq data upload, followed by automated quality control, adapter trimming, and genome alignment. The NovelScan module identifies novel transcripts, including lncRNAs and mRNAs, and characterizes their expression levels, lengths, exon counts, conservation, and coding potential. The InfoScan software is available for download at https://infoscan-docs.readthedocs.io/en/latest/index.html (accessed on 1 July 2023).

### 2.2. InfoScan Reveals the Abundance of Unannotated lncRNAs in GBM

**Figure 2:**
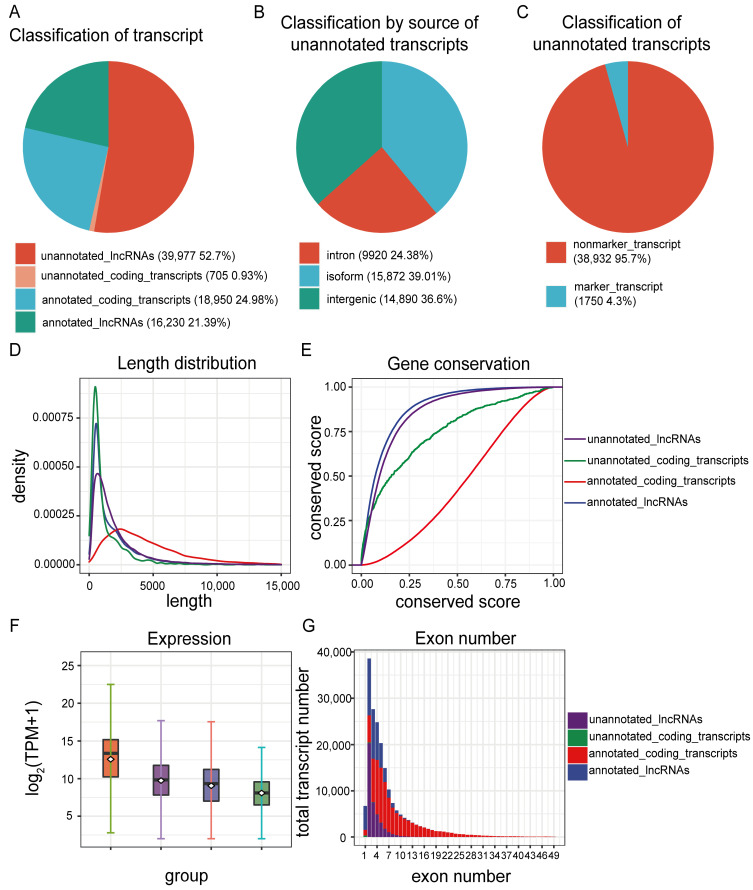
Characteristics of unannotated transcripts in GBM. (**A**) Distribution of identified transcript types and their respective quantities in GBM. (**B**) Classification and abundance of unannotated transcripts, categorized as novel isoforms, intergenic-derived, and intronic-derived transcripts. (**C**) Categorization of unannotated transcripts into marker and non-marker transcripts, along with their abundances. (**D**) Density plot illustrating the length distribution of transcripts. (**E**) Box plot showing the average expression levels of transcripts. (**F**) Cumulative distribution plot indicating the conservation levels of transcripts across species. (**G**) Transcriptional map displaying the exon count distribution of the identified transcripts.

The InfoScan platform was used to analyze full-length scRNA-seq data from the tumor and adjacent nontumor tissues of four GBM patients [24]. After quality control, 75,862 transcripts were identified across 3589 cells, including 39,977 (52.7%) unannotated lncRNAs (Figure 2A). These transcripts were classified into three categories: intergenic regions, intronic regions, and novel splicing variants, with splicing variants being the most abundant (Figure 2B).

Using the NovelScan module, further analysis revealed that these unannotated transcripts were mostly lncRNAs, tended to be shorter, had fewer exons, and presented lower conservation across species (Figure 2D–G). On the basis of their sequence characteristics and expression levels, we further filtered 1750 unannotated lncRNAs that exhibited robust cell type specificity as marker transcripts (Figure 2C). These findings indicate that the tissue/cell-specific expression patterns of lncRNAs contribute to the existence of a substantial reservoir of unannotated lncRNAs within organisms, which remains to be fully explored.

### 2.3. Unannotated lncRNAs Mark a Neoplastic-Stemness Subpopulation

**Figure 3:**
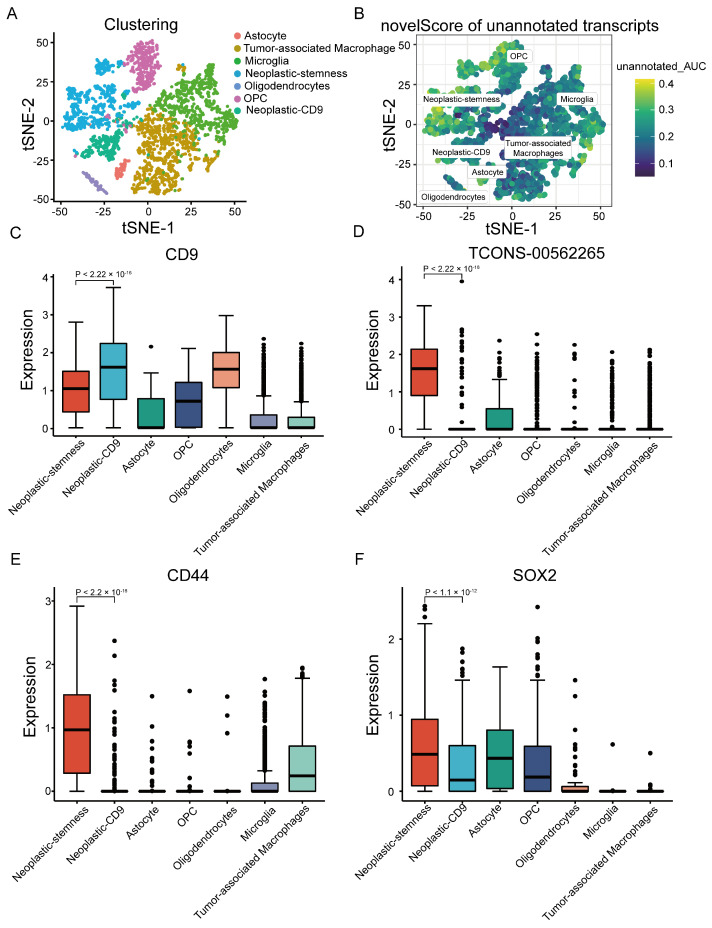
Expression of unannotated LncRNAs across different cell clusters. (**A**) t-SNE map illustrating the identification of six distinct cell types: astrocytes, tumor-associated macrophages, microglia, neoplastic cells, oligodendrocytes, and oligodendrocyte precursor cells (OPCs). (**B**) t-SNE map displaying the novelScore of unannotated lncRNAs derived from full-length scRNA-seq data. (**C**–**F**) Boxplots depicting the enrichment scores of unannotated lncRNAs across various cell types. Significant expression differences between neoplastic-stemness and neoplastic -CD9 subgroups are shown for (**C**) CD9, (**D**) TCONS-00562265, (**E**) CD44, and (**F**) SOX2.

To investigate the distribution of unannotated lncRNAs across cell populations, a novelScore metric was introduced to assess transcript specificity within six major GBM cell types. Based on the novel score, neoplastic cells were subdivided into two subgroups: neoplastic-sub1 and neoplastic-sub2. Notably, neoplastic-sub1, enriched with unannotated lncRNAs, exhibited distinct stem cell characteristics, and was designated neoplastic-stemness (Figure 3A,B and Figure S1A,B). In contrast, neoplastic sub2 showed significant upregulation of CD9, leading to its designation as neoplastic-CD9 (Figure 3C and Figure S1C).

A substantial number of novel lncRNAs were enriched in neoplastic-stemness cells, including TCONS-00562265, which was highly expressed in this subgroup but minimally detected in neoplastic-CD9 cells.(Figure 3D and Figure S1D). Coexpression analysis via the FunScan module revealed that CD44 and SOX2, well-known stem cell markers, were significantly coexpressed with TCONS-00562265, emphasizing the stem-like properties of neoplastic-stemness (Figure 3E,F and Figure S1E,F). Additionally, TCONS-00562265 exhibited coexpression with key genes involved in the epithelial-to-mesenchymal transition (EMT) pathway (Figure S2), suggesting its potential role in tumor metastasis and cancer stem cell regulation.

The functional classification of TCONS-00562265 as a non-coding RNA was confirmed through CPAT, RNAsamba, and LncFinder, with minimal ribosomal coverage observed in UCSC [29] ribosome profiling data (Figure S3A). Despite lacking protein-coding potential, RBPmap [30] analysis identified binding sites for key RNA-binding proteins (RBPs) implicated in GBM (Figure S3B), including IGF2BP, ELAVL4, and MSI1, which regulate processes such as RNA splicing, stability, and tumor cell self-renewal [31,32,33]. These interactions suggest that TCONS-00562265 may influence glioblastoma progression by modulating RBP activity to maintain cancer cell stemness. 

### 2.4. Validation of Neoplastic-Stemness Stem Cell Characteristics Through Integrated InfoScan, Enrichment Analysis, and Spatial Transcriptomic Profiling

**Figure 4:**
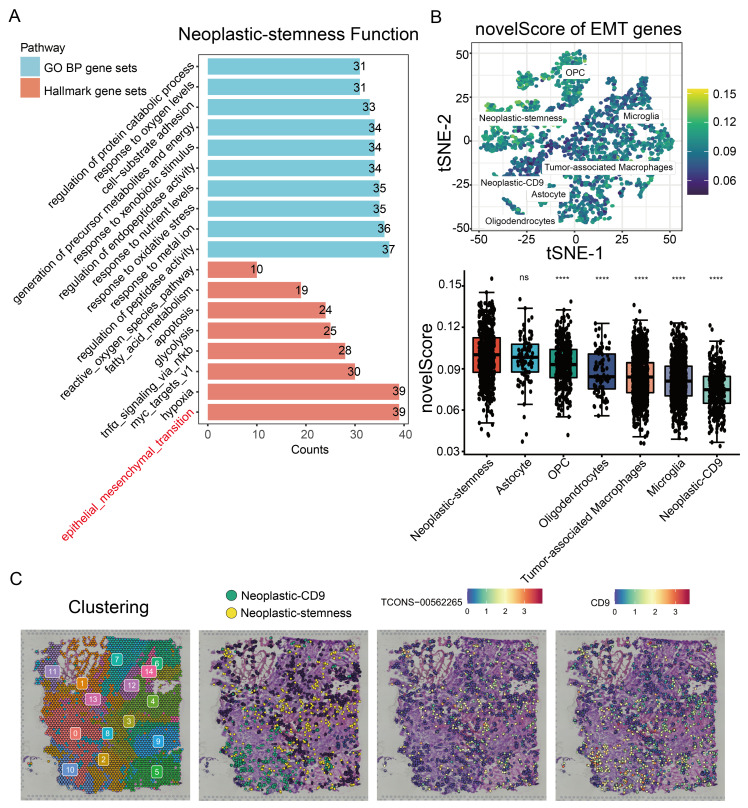
Neoplastic-stemness exhibits stem cell characteristics. (**A**) Pathway enrichment bar chart for Hallmark and GO biological process (BP) gene sets associated with neoplastic-stemness marker genes. The y-axis represents pathway terms, while the x-axis indicates the number of genes enriched in each term. (**B**) The top panel displays the novelScore for the EMT gene set mapped onto the t-SNE plot, while the bottom panel presents a boxplot of the novelScore for the EMT gene set (**** *p* < 0.0001 indicates a significant difference, ns indicates not significant). (**C**) Spatial transcriptomics data of GBM illustrating the spatial distribution of two cell subtypes: neoplastic-stemness (yellow) and neoplastic-CD9 (green). The expression and distribution of TCONS-00562265 and CD9 in GBM tissue sections are shown, with higher expression levels indicated by deeper red colors.

Differential expression analysis between the neoplastic-stemness and neoplastic-CD9 subgroups was performed using the InfoScan platform, followed by gene ontology (GO) and hallmark gene set enrichment analyses of the DEGs. Consistent with the coexpression analysis of unannotated lncRNA TCONS-00562265, highly expressed genes in neoplastic-stemness cells were enriched in pathways related to migration and invasion, such as EMT and hypoxia signaling (Figure 4A).

In addition, identified cells with active EMT signatures using gene sets from the EMTome database (Figure 4B) [34]. These results revealed significant enrichment of EMT-related genes in neoplastic-stemness cells, reinforcing their migratory capacity and stemness characteristics.

The CellInfo module of InfoScan, which extends CytoTRACE [35], was applied to assess the differentiation potential of neoplastic-stemness cells on the basis of gene expression. Neoplastic-stemness cells exhibited the highest differentiation potential, indicating their identity as cancer stem cells with notable differentiation abilities (Figure S4A,B). 

To explore the spatial distribution of neoplastic-stemness cells in GBM tissue, the SpatialScan module of InfoScan was applied. Unsupervised clustering partitioned the tissue into 15 regions, annotated by full-length scRNA-seq data. Neoplastic-CD9 cells were localized primarily in regions 0 and 10, while neoplastic-stemness cells were broadly distributed throughout the GBM tissue (Figure 4C). This distribution pattern suggests that the widespread presence of neoplastic-stemness cells plays a crucial role in tumor progression [36]. Additionally, TCONS-00562265 was extensively expressed in areas outside regions 0 and 10, where CD9 was predominantly observed (Figure 4C). 

The novelScore was calculated from full-length scRNA-seq data to identify cells with active EMT signatures via gene sets from the EMTome database (Figure 4B) [37]. The results revealed significant enrichment of EMT-related genes in neoplastic-stemness cells, reinforcing their migratory capacity and stemness characteristics.

The CellInfo module of InfoScan, which extends CytoTRACE [38], was applied to assess the differentiation potential of neoplastic-stemness cells on the basis of gene expression levels. This analysis revealed that neoplastic-stemness cells presented the highest differentiation potential among the identified subtypes (Figure S4A,B), indicating their possible identity as cancer stem cells with substantial differentiation capabilities.

### 2.5. Neoplastic-Stemness Significantly Increased in Large Cohorts of GBM Patients

To investigate the characteristics of neoplastic-stemness in a large-scale cohort, the CellInfo module of InfoScan was utilized to estimate cell type proportions in GBM samples across broader patient datasets, which include the TCGA (175 samples) and CGGA (325 samples) databases.

**Figure 5:**
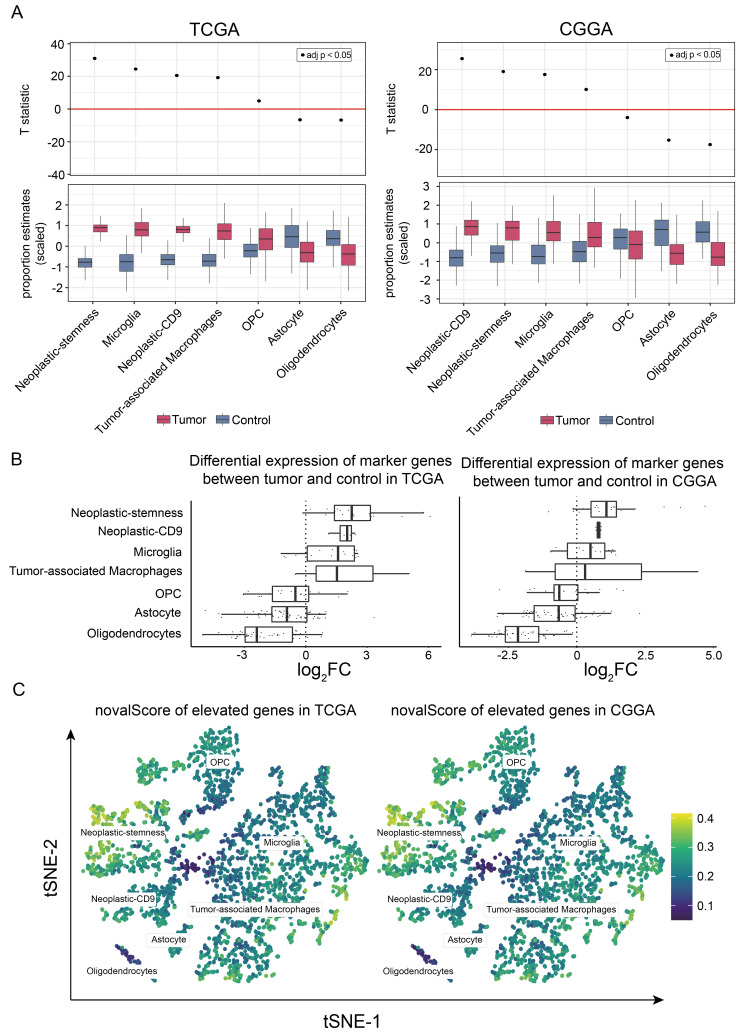
Distribution of neoplastic-stemness in GBM samples. (**A**) Cell type abundances between tumor and normal samples in bulk RNA sequencing data from the TCGA and CGGA databases. The upper section presents T statistics from the paired *t*-test and adjusted *p*-values calculated using the false discovery rate (FDR) based on the paired Wilcoxon test. The lower section displays a bar chart illustrating estimated cell type proportions categorized by tissue type (tumor and control). (**B**) Fold change of cell type marker genes in bulk RNA sequencing data. The y-axis represents different cell types, while the x-axis indicates the fold change of marker genes between tumor and normal samples. (**C**) The UMAP plot shows the novelScore of tumor-upregulated marker genes from the TCGA and CGGA cohorts.

Using marker proteins identified through single-cell RNA sequencing as diagnostic signatures (Table S1), we conducted an analysis to assess the variations in the cellular composition between neoplastic and normal tissues within TCGA dataset. The results revealed a significant increase in the abundance of neoplastic-stemness, neoplastic-CD9, microglia, and tumor-associated macrophages in tumor tissues compared to normal brain tissues, with the most pronounced increase in neoplastic-stemness (Figure 5A).

A similar analysis of 325 GBM samples from the CGGA database, which included 222 high-grade gliomas classified as WHO grades III and IV, mirrored the TCGA findings, confirming a substantial increase in neoplastic-stemness proportions in tumor samples relative to normal tissues (Figure 5A).

Subsequent analysis of marker gene expression between tumor and normal samples revealed that marker genes associated with neoplastic-stemness presented the greatest average log-fold changes in both the TCGA and CGGA datasets (Figure 5B). These genes demonstrated strong coexpression in bulk RNA sequencing data and correlated significantly with their respective cell proportions (Figure S5A,B). Gene-level analysis confirmed a substantial increase in neoplastic-stemness cell types in tumor samples compared with normal samples. The novelScore was also calculated via full-length scRNA-seq data to identify cells with significantly upregulated gene activity in tumor samples (Figure S6A–D). These genes were significantly enriched in neoplastic-stemness cells, reinforcing their critical role in GBM pathology.

Collectively, these findings demonstrate a significant increase in neoplastic-stemness in tumor samples, which is associated with higher GBM malignancy. Validation in two independent large cohorts confirmed the reproducibility and reliability of InfoScan, highlighting its potential as a tool for advancing cancer research.

### 2.6. Increased Proportion of Neoplastic-Stemness Correlates with Reduced Survival in GBM Patients

**Figure 6:**
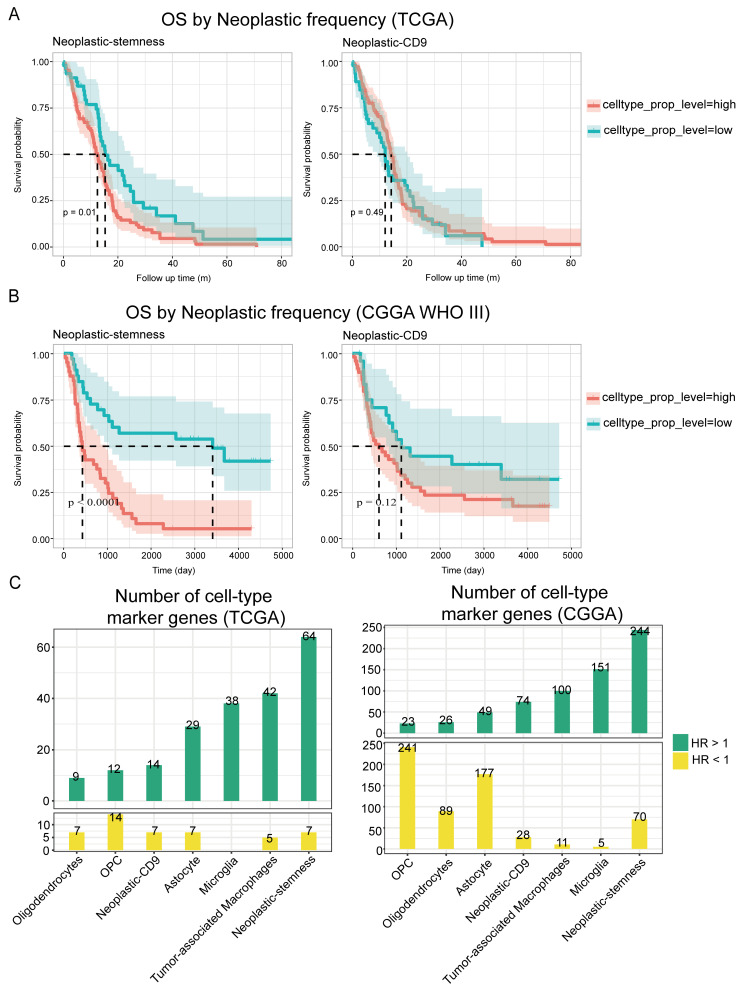
Association between neoplastic-stemness and survival in GBM patients. (**A**) Overall survival curves for neoplastic-stemness (**left**) and neoplastic-CD9 (**right**) in patients from the TCGA cohort. (**B**) Overall survival curves for neoplastic-stemness (**left**) and neoplastic-CD9 (**right**) in patients with WHO type III gliomas from the CGGA cohort. The dashed line indicates the median survival time. (**C**) The number of genes with significant hazard ratios (HR) in the TCGA (**left**) and CGGA (**right**) databases, respectively. The x-axis represents cell types, while the y-axis indicates the number of genes, with green indicating HR > 1 and yellow indicating HR < 1.

To investigate the impact of neoplastic-stemness cells on overall survival in GBM patients, the CellInfo module of InfoScan was utilized to analyze samples from the TCGA database. Initial assessments combining neoplastic-stemness and neoplastic-CD9 as a single cluster indicated no significant correlation with patient survival (Figure S7). Further evaluations revealed that patients with higher neoplastic-stemness proportions had significantly lower survival rates (*p* = 0.01; Figure 6A), while neoplastic-CD9 did not affect survival (Figure 6A). Proportions of other cell types had no impact on survival (Figure S8A). Validation with a Cox proportional hazards regression model confirmed these findings, revealing a significantly increased hazard ratio (HR) for patients with high neoplastic-stemness proportions (*p* = 0.006; Figure S8B).

In the CGGA database, which includes gliomas of WHO grades III and IV, high neoplastic-stemness proportions were linked to reduced survival across both grades (Figure 6B, Figures S9A and S10A). Cox regression analysis revealed an increased HR for the high neoplastic-stemness group (HR = 2.48, *p* = 0.002; Figure S9B), which remained significant after adjusting for pathological stage (HR = 2.31, *p* = 0.003; Figure S10B). In WHO grade IV patients, a high proportion of neoplastic-stemness was a significant factor for reduced survival. These results, consistent across both cohorts, underscore the critical role of neoplastic-stemness in glioblastoma progression.

Further analysis of risk genes in GBM cell types revealed that neoplastic-stemness cells contained the greatest number of significant risk genes: 64 in the TCGA cohort and 244 in the CGGA cohort. In contrast, oligodendrocyte precursor cells (OPCs) had the highest number of significant protective genes (HR < 1, *p* < 0.05) (Figure 6C). Univariate Cox regression analysis across all samples identified genes linked to poor survival outcomes, which were notably enriched in neoplastic-stemness cells (Figure S11). These findings validate InfoScan’s ability to uncover biologically and clinically critical subpopulations, emphasizing its potential for multi-cohort validation and risk gene prioritization in malignancies.

### 2.7. Tumor Microenvironment Induce Neoplastic-Stemness via the SPP1–CD44 Axis

**Figure 7:**
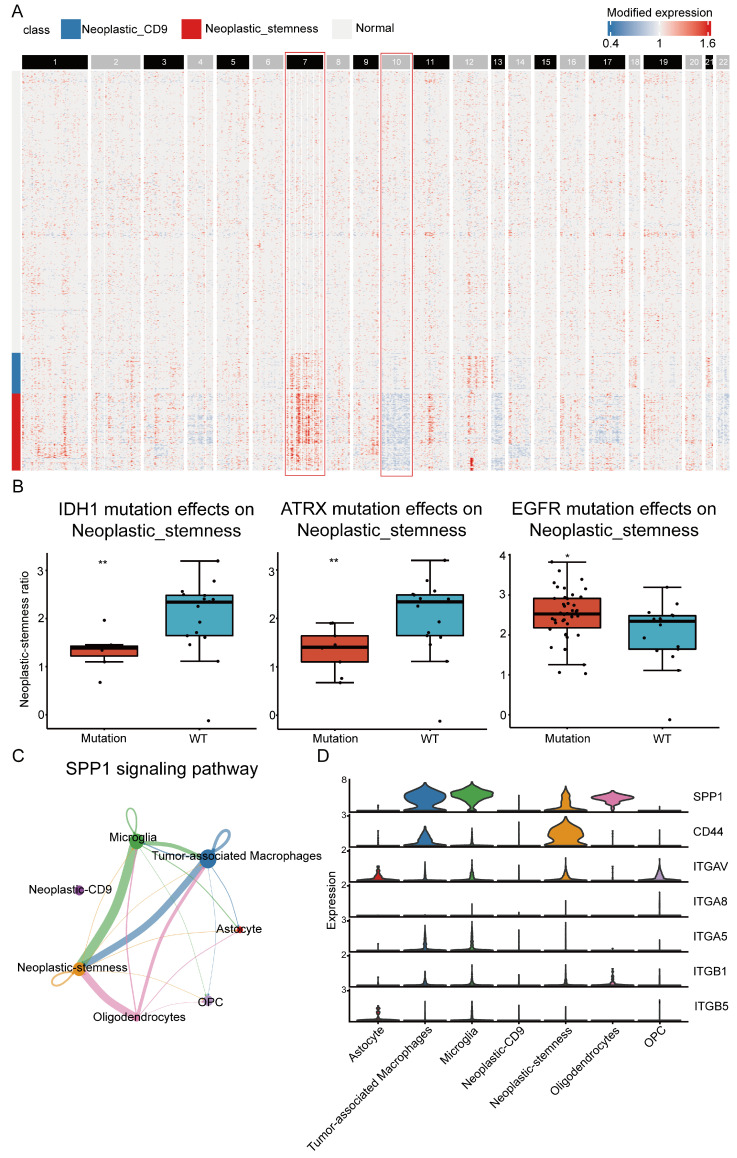
Genomic characteristics and intercellular communication of neoplastic-stemness. (**A**) The heatmap illustrates relative gene expression levels across chromosomes 1 to 22, arranged sequentially from left to right. Regions of gene overexpression are marked in red, while areas of gene underexpression are marked in blue. Neoplastic cells showed significant amplifications of chromosome 7 and deletions of chromosome 10 (red box). (**B**) Boxplots display the neoplastic-stemness ratio in samples with WT and *IDH1*, *ATRX*, *EGFR* gene mutation samples (* *p* < 0.05, ** *p* < 0.01). (**C**) The circular diagram depicts the distribution of the SPP1 signaling pathway across various cell types. The diameter of each circle represents the cell count within each cell type, while the thickness of the connecting lines between circles indicates the relative proportion of SPP1 signaling interactions between the respective cell types. (**D**) Expression levels of each receptor and ligand involved in the SPP1 signaling pathway across different cell types.

To explore the mechanisms driving neoplastic-stemness, the CellInfo module analyzed copy number variations (CNVs) across cell subpopulations using full-length scRNA-seq data. Neoplastic cells showed significant amplifications of chromosome 7 and deletions of chromosome 10, with these aberrations more pronounced in the neoplastic-stemness group than in the neoplastic-CD9 group (Figure 7A). Previous studies have linked these CNVs to worse clinical outcomes in IDH wild-type glioblastoma [39], suggesting a key role of CNVs in neoplastic-stemness formation and its association with poor prognosis.

To investigate the molecular mechanisms, InfoScan analyzed somatic mutations in TCGA GBM samples. Three frequently mutated genes—*IDH1*, *ATRX*, and *EGFR*—were significantly associated with neoplastic-stemness. In *IDH1* and *ATRX* mutant samples, the proportion of neoplastic-stemness cells was reduced, while *EGFR* mutations increased this proportion (Figure 7B). *ATRX* mutations, often co-occurring with *IDH1* mutations, are typically linked to better prognosis, reinforcing that greater neoplastic-stemness correlates with poorer outcomes.

Further analysis of differentially expressed genes between wild-type (WT) and mutant samples revealed that genes downregulated in IDH1 mutants and upregulated in EGFR mutants were enriched in neoplastic-stemness cells, while those downregulated in ATRX mutants were found mainly in tumor-associated macrophages (TAMs) (Figure S12). This suggests that TAMs in the tumor microenvironment may activate neoplastic-stemness through cell-to-cell interactions, driving tumor progression.

The CellInfo module also analyzed communication between neoplastic-stemness cells and other tumor microenvironment cells, revealing critical interactions mediated by the SPP1–CD44 axis. Neoplastic-stemness macrophages interacted significantly with TAMs, microglia, and oligodendrocytes (Figure 7C). The SPP1-CD44 interaction was identified as the most influential in cancer progression, with SPP1 highly expressed in TAMs and CD44 overexpressed in neoplastic-stemness cells (Figure 7D). SPP1 has been shown to activate downstream pathways like PI3K/AKT and Rho family small GTPases, enhancing tumor cell adhesion, migration, and invasion [40]. Inhibiting these pathways could be an effective strategy for targeting patients with high neoplastic-stemness [41].

### 2.8. The Therapeutic Effects of Omipalisib on Neoplastic-Stemness Cells

**Figure 8:**
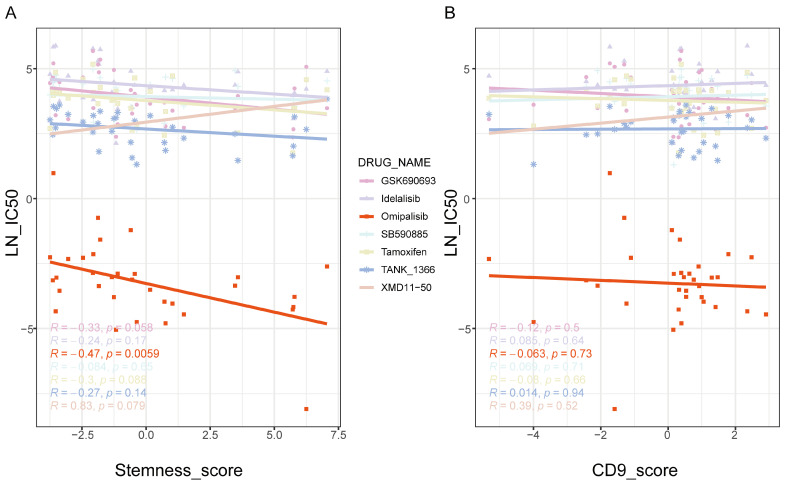
Scatter plots showing the correlation between IC50 values and cell characteristic scores under different drug treatment conditions. (**A**) The scatter plot illustrates the correlation between the Stemness_score and the efficacy of various drugs, represented by distinct colors. Each point corresponds to a specific drug, with its position indicating the IC50 value relative to the Stemness_score. The R value within the figure denotes the correlation coefficient, indicating both the strength and direction of the relationship between drug potency and the Stemness_score. The *p* value is provided to indicate the statistical significance of each correlation. (**B**) CD9_score: Scatter plot displaying the correlation between the CD9_score and IC50 values across various drug treatments.

Based on the hypothesis that PI3K/AKT pathway inhibitors may target neoplastic-stemness cells, gene expression data and drug sensitivity (IC50 values) for GBM cell lines were obtained from the Cancer Drug Sensitivity in Genomics (GDSC) database [42]. A stemness score was established using the expression levels of neoplastic-stemness marker genes, and its correlation with IC50 values across various drug treatments was assessed. The results revealed a significant negative correlation between the Stemness_score and omipalisib sensitivity, indicating that cells with higher stemness scores are more sensitive to omipalisib (Figure 8A).

As expected, omipalisib, a selective PI3K inhibitor, effectively suppresses PI3K/AKT pathway activation mediated by SPP1 and CD44 interaction [43]. These findings suggest enhanced therapeutic efficacy of omipalisib in cancer cells with prominent stemness properties. In contrast, the CD9 score, calculated similarly, showed no significant correlation with patient survival outcomes, and cells with higher CD9 scores did not exhibit increased sensitivity to omipalisib (Figure 8B). Further application of the Stemness_score to GBM patient samples showed that higher stemness scores correlated with poorer prognosis (Figure S13A,B), indicating that omipalisib could offer precision therapy for these patients, potentially improving treatment outcomes.

### 2.9. Application of InfoScan in Breast Cancer Data Reveals Its Generalizability

The generalizability of InfoScan was demonstrated through its application to breast cancer data, further validating its utility across various disease types. Single-cell RNA-seq data from breast cancer samples under hypoxic conditions were obtained from a publicly available dataset [44], comprising 384 cells categorized into hypoxia (Hypo) and normal (Norm) groups. Using InfoScan, 5511 unannotated lncRNAs were identified, with 17 upregulated and 33 downregulated lncRNAs detected in the hypoxic group after applying selection criteria (average log2 fold change > 1, adjusted *p*-value < 0.05).

**Figure 9:**
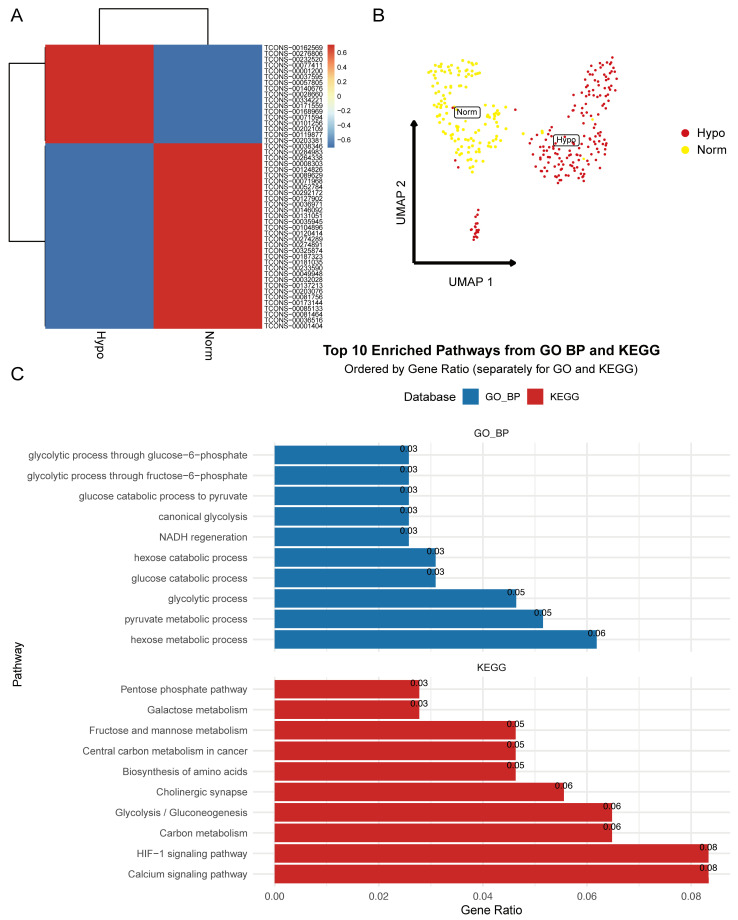
Application of InfoScan in breast cancer data. (**A**,**B**) shows the analysis of differential expression and clustering of lncRNAs under hypoxic and normal conditions. (**C**) Functional enrichment analysis of upregulated lncRNAs in the hypoxic group. Enriched pathways are associated with tumor cell proliferation, invasion, and metabolic reprogramming, including glycolysis, NADH regeneration, and carbon metabolism.

To explore the biological functions of these lncRNAs, clustering and functional enrichment analyses were performed using GO and KEGG databases. Comparative analysis of lncRNA expression revealed significant alterations under hypoxic conditions (Figure 9A), corroborated by UMAP dimensionality reduction analysis, which clearly separated the two groups based on lncRNA expression profiles (Figure 9B). Functional enrichment analysis showed that upregulated lncRNAs were linked to pathways involved in tumor cell proliferation and invasion, such as glycolysis, NADH regeneration, and carbon metabolism, critical for metabolic reprogramming in low oxygen environments. These lncRNAs likely facilitate glycolytic flux and enhance survival under hypoxic stress (Figure 9C).

Conversely, downregulated lncRNAs were associated with pathways involved in cell migration, chromosome segregation, nuclear division, and cell cycle regulation, reflecting a diminished proliferative capacity under oxygen deprivation. These downregulated lncRNAs also participated in tumor cell adaptation mechanisms, influencing migratory capabilities under hypoxic stress (Figure S14).

These findings validate InfoScan’s potential in cancer data analysis, showing how the hypoxic microenvironment regulates lncRNA expression to affect tumor biology.

## 3. Discussion

This study employed single-cell sequencing and the InfoScan visualization tool to identify a rare neoplastic-stemness cell type expressing specific lncRNAs in GBM, associated with poor prognosis. The TME secreted SPP1, binding to CD44 on neoplastic-stemness cells, activating the PI3K/AKT pathway and promoting lncRNA transcription. Previous studies have shown that this pathway activates transcription factors to induce lncRNA expression [45], though further validation is needed to clarify its role in tumor invasion, migration, angiogenesis, and immune evasion.

Dimensionality reduction clustering using only lncRNAs effectively distinguished neoplastic-stemness cells from neoplastic-CD9 cells, though it was less effective in differentiating microglia from TAMs (Figure S15A,C). In contrast, annotated mRNA transcripts clearly separated microglia and TAMs but failed to distinguish between neoplastic-stemness and neoplastic-CD9 cells (Figure S15C,D). This analysis highlighted that lncRNAs excelled in classifying cancer cell populations, while annotated transcripts were better for immune cell classification (Figure S15B). These results underscore the utility of unannotated lncRNAs in detecting rare cancer cell subsets, facilitating the discovery of overlooked cell subtypes in cancer progression.

The SPP1-CD44 axis plays a crucial role in the maintenance and progression of glioblastoma stem cells, influencing tumor aggressiveness and therapeutic resistance. SPP1, a secreted glycoprotein, binds to the CD44 receptor on cancer cells, activating key pathways like PI3K/AKT, FAK, and NF-κB [37,38]. This interaction promotes cell migration, invasion, and survival, maintaining stem-like properties linked to poor GBM prognosis. Additionally, SPP1 recruits immunosuppressive myeloid cells, further promoting tumor progression [46,47]. Elevated SPP1 expression in TME-associated cells and CD44 upregulation in neoplastic-stemness cells suggests a similar mechanism in GBM. These findings support the role of SPP1-CD44 signaling in promoting cancer stem cell traits, including self-renewal, drug resistance, and immune evasion [40]. Targeting this axis may disrupt GBM stemness and improve treatment efficacy. Future studies should explore combined therapies, like PI3K inhibitors and SPP1-CD44 blockade, to better target neoplastic-stemness cells and overcome GBM resistance mechanisms. Similar roles for the SPP1-CD44 axis have been observed in other cancers, such as pancreatic cancer, where SPP1 secreted by cancer-associated fibroblasts (CAFs) enhances stem cell characteristics through CD44 interaction [21]. These insights highlight the importance of the TME in regulating tumor stem cell properties, suggesting potential therapeutic targets.

The application of PI3K inhibitors in GBM is actively researched, as the PI3K/AKT/mTOR pathway is often hyperactivated in GBM and critical for tumor growth, survival, migration, and invasion [48]. Although Omipalisib shows potential against neoplastic-stemness cells in GBM, several challenges exist, including parallel signaling pathways that may reduce the effectiveness of PI3K inhibition [49]. Genetic alterations like PTEN, EGFR, or PDGFRA can lead to resistance [50,51] ], while off-target effects and toxicity limit the use of PI3K inhibitors in clinical trials [52]. The TME’s heterogeneity, with immunosuppressive components and hypoxic regions, favors cancer stem cell survival after PI3K blockad [53]. Combining PI3K inhibitors with radiation, temozolomide, or immune-modulating agents is being explored to enhance outcomes [54,55]. Preliminary studies suggest that combining buparlisib with standard therapies may yield synergistic effects, though clinical data are limited [56]. Identifying predictive biomarkers for PI3K inhibitor sensitivity could guide more personalized treatment approaches. Overcoming resistance and minimizing off-target effects will be key for clinical success.

Using InfoScan, researchers can identify rare cell subtypes in cancers by analyzing unannotated lncRNAs, opening new avenues for cancer treatment. This approach also aids in discovering novel transcripts that improve cell type marker identification, enhancing therapeutic targeting. Experimental validation is needed to confirm the role of these transcripts in glioma stem cells. Future plans aim to enhance InfoScan for compatibility with third-generation sequencing, enabling the identification of a broader range of novel transcripts at the single-cell level.

However, bioinformatic predictions have limitations. Correlation-based analyses suggest potential functions but do not prove causation. Single-cell transcriptomic data may also suffer from technical biases, such as dropouts and batch effects. Thus, rigorous experimental validation is necessary to confirm the roles of unannotated lncRNAs. Experiments like in vitro knockdown or overexpression studies of TCONS-00562265 can assess its impact on cell viability, self-renewal, and invasion. Additionally, assessing the functional relationship between SPP1-CD44 signaling and neoplastic-stemness cells through targeted inhibition and rescue experiments will establish clearer causality. These experimental approaches are essential to translate InfoScan findings into clinically relevant targets.

## 4. Materials and Methods

### 4.1. Data Source

Full-length scRNA-seq data were obtained from the GSE84465 dataset, containing 3589 cells from tumor and adjacent nontumor tissues [57]. Spatial transcriptomic data for GBM were sourced from 10× Genomics (available at https://www.10xgenomics.com/ (accessed on 19 October 2014)). Additional data included 170 tumor and 5 paracancerous samples from the TCGA GBM cohort [58], and 325 glioma and 20 nontumor samples from the CGGA cohort [59]. Normal brain tissues were sourced from GTEx (https://www.gtexportal.org (accessed on 23 October 2013)). Somatic mutation data were acquired from exome sequencing by the Genome Data Analysis Center at the Broad Institute (http://gdac.broadinstitute.org (accessed on 17 May 2002)), and the glioblastoma-specific EMT signature came from the EMTome database [34].

### 4.2. Genome Mapping and Transcript Assembly

Quality control of the full-length scRNA-seq data was performed with Fastp (v0.12.4) [60], followed by genome alignment via HISAT2 (v2.2.1) [61] and transcript assembly via StringTie2 (v2.1.7) [62]. Cuffcompare (v2.2.1) [63] was used for comparative analysis, and coding potential was predicted with CPAT (v3.0.4) [64], RNAsamba (v0.2.5) [65], and LncFinder (v1.1.4) [66]. Conservation analysis was performed via bigWigAverageOverBed (v2) [67]. Transcripts were reassembled using StringTie2 [62] and compared to reference genome annotations via Cuffcompare. Unannotated transcripts were categorized under class codes ‘u’, ‘i’, and ‘j’. Coding potential was assessed, and transcripts identified by all three tools as coding, with sufficient expression levels, were classified as unannotated coding transcripts. Noncoding transcripts, exceeding 200 nucleotides and meeting expression criteria, were designated as unannotated lncRNAs.

### 4.3. Cell Annotation and Spatial Transcriptome Analysis

Single-cell data were pooled together and analyzed with Seurat (v4.1.1) [68], annotated with SingleR (v1.8.1) and CellTypist (v1.2.0) [69], and visualized via ggplot2 (v3.3.6) [70], whereas report generation was facilitated via the Rmarkdown package (v2.14) [71]. Cell-type specificity was quantified with cummeRbund (v2.36.0). Heatmaps were generated using ComplexHeatmap (v2.10.0). The CytoTRACE package (v0.3.3) [35] assessed differentiation potential, and BisqueRNA (v1.0.5) [72] was used for transcriptome decomposition. Spatial transcriptome data were analyzed with Seurat (v4.1.1) and integrated with CellTrek (v0.0.94) for single-cell and spatial transcriptome datasets.

### 4.4. Gene Set Scoring

Single-cell data scoring based on lncRNA markers was performed using the AUCell package (v1.16.0). Genes in each cell were ranked by expression levels, and enrichment of genes in the novel lncRNA marker gene set G, termed the novelScore, was calculated with the AUC formula:

$$AUC\left(G\right)=\int_{0}^{1}TPR\left(f\right)dFPR\left(f\right),$$

where TPR(f) represents the proportion of genes from set G in the top f% of expressed genes and FPR(f) represents the proportion of non-G genes in the same top f%. Higher AUC values indicate greater enrichment of the gene set. The AUCell_exploreThresholds function was used to establish varying AUC thresholds for selective screening based on lncRNA marker activity.

### 4.5. Evolutionary Conservation Analysis

The phastCons score, ranging from 0 to 1, quantifies base conservation across species [73]. Average phastCons scores for human and mouse exons were calculated using data from 100 vertebrate species in UCSC Genome Browser. These scores provide conservation probabilities for each genomic position. The mean phastCons score for each transcript was computed, excluding null values, with the bigWigAverageOverBed tool (v2). The empirical cumulative distribution function (ECDF) was visualized using ggplot2.

### 4.6. Tissue- or Cell-Specific Analysis

Tissue or cell specificity of transcripts was quantified using Jensen–Shannon divergence [74]. Mean expression levels across tissues or cell types were calculated, and specificity was assessed using the cummeRbund package. The ECDF of these scores was visualized using the stat_ecdf function, and average expression levels were displayed with the ComplexHeatmap package.

### 4.7. Gene Coexpression and Enrichment Analysis

Gene coexpression was analyzed via Pearson correlation, with *p* values adjusted using the FDR method. Genes with *p* < 0.05 were considered coexpressed with the target transcript. Pathway enrichment was performed using gene sets from MSigDB (v7.5.1) [75]. GO enrichment was assessed with the enrichGO function, covering 10,532 gene sets. KEGG pathway analysis was done with enrichKEGG, encompassing 186 pathways. Hallmark gene set enrichment was conducted using Enricher for 50 biological states or processes.

### 4.8. Cell Communication Analysis

Cell communication was analyzed with the CellChat package (v1.1.0) [76], categorizing signaling pathways and identifying conserved or context-specific pathways. Gene expression data and cell labels, along with the human ligand–receptor database, were used. Overexpressed ligands/receptors in specific cell groups were mapped onto a protein–protein interaction (PPI) network to identify significant signaling interactions.

### 4.9. Estimating Cell Type Proportions from Bulk GBM Data

Cell type proportions in bulk RNA sequencing from the TCGA GBM cohort were estimated using the bisulfite method [73], with cell-specific markers identified from single-cell data. PCA of normalized transcriptome data was applied, identifying seven major cell types for analysis. Analyses were extended to the CGGA dataset, excluding low-grade glioma samples, with paired t tests comparing tumor and nontumor cell proportions. DESeq2 was used for differential expression analysis, identifying upregulated genes with padj < 0.05 and log2FoldChange > 1, and downregulated genes with padj < 0.05 and log2FoldChange < −1.

### 4.10. Survival Analysis

Survival analysis was conducted using the survival package (v3.3.1), assessing the survival function S(t)=P(T>t). Differential expression was analyzed with DESeq2 (v1.34.0), and pathway enrichment with clusterProfiler (v4.2.2) [77]. Cox regression models evaluated the impact of cell type proportions on survival, stratifying patients into high- and low-cell-type groups. Kaplan–Meier survival curves were generated. Gene expression was correlated with survival, with univariate regression identifying genes linked to survival based on hazard ratios (HR). The AUCell method was used for gene scoring in single-cell data.

### 4.11. Somatic Mutation Analysis

Somatic mutation data for the TCGA-GBM cohort were obtained from exome sequencing. Tumor samples with mutations (missense, nonsense, frameshift, or synonymous) were classified as mutation-bearing. Paired t tests compared cell proportion estimates between mutation-bearing and wild-type samples. Pathway enrichment was conducted for genes affecting cell type proportions.

## 5. Conclusions

Single-cell sequencing identifies rare cancer cell types via specific marker genes and lncRNAs, but analytical tools are limited. InfoScan, a cross-platform visualization tool, was developed to detect novel lncRNAs from full-length single-cell sequencing data and analyze their enrichment in specific cell types. Using InfoScan, a rare GBM cell population with numerous lncRNAs and high stemness, termed neoplastic-stemness, was identified. This population was linked to poorer survival outcomes. Cell communication analysis suggested that SPP1 secretion by tumor cells binds to CD44 on neoplastic stem cells, activating the PI3K/AKT pathway and enhancing lncRNA transcription. These lncRNAs regulate tumor behaviors such as invasion, migration, angiogenesis, and immune evasion. PI3K inhibition significantly reduced neoplastic-stemness cell activity. A stemness score was developed to identify GBM patients with poor prognosis who could benefit from PI3K inhibitors like omipalisib. This work offers a tool for identifying rare cell types and analyzing lncRNA functions, improving the understanding of tumor heterogeneity and progression and supporting personalized treatment strategies based on molecular signatures.

## Data Availability

Public data utilized in this study can be accessed in Section 4. The InfoScan software is available for download at: https://infoscan-docs.readthedocs.io/en/latest/index.html (accessed on 1 July 2023).

## Supplementary Materials

The following supporting information can be downloaded at: https://www.mdpi.com/article/10.3390/ijms26052208/s1.

## Abbreviations

The following abbreviations are used in this manuscript:

GBM	Glioblastoma multiforme
TME	Tumor microenvironment
lncRNA	Long non-coding RNA
scRNA-seq	Single-cell RNA sequencing
EMT	Epithelial–mesenchymal transition
CAF	Cancer-associated fibroblasts
