# Supplementary material for: InfoScan: A New Transcript Identification Tool Based on scRNA-Seq and Its Application in Glioblastoma
Source: Int J Mol Sci. 2025 Feb 28;26(5):2208. doi: 10.3390/ijms26052208 (PMC11900204; doi:10.3390/ijms26052208)
Supplement: Supplementary file 1 [file ijms-26-02208-s001.zip › Supplementary.pdf]

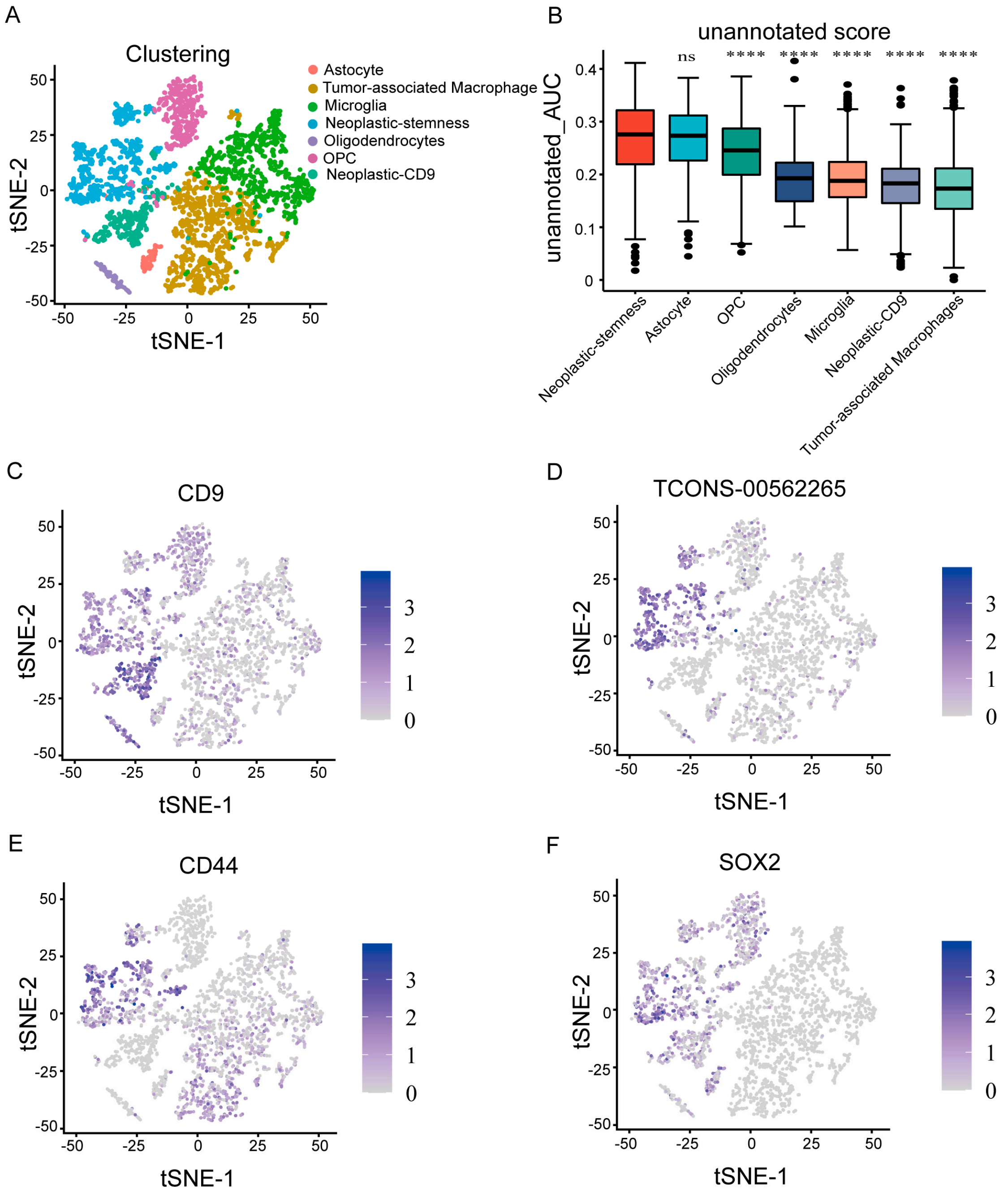

Supplementary Figure S1 | (A) t-SNE map illustrating the identification of six distinct cell types: astrocytes, TAM, microglia, neoplastic-stemness, neoplastic, oligodendrocytes, and oligodendrocyte precursor cells (OPCs). (B) The boxplot shows the enrichment score of unannotated transcripts in different cell types. The asterisk indicates that there is a significant difference in the enrichment fraction between neoplastic-novel and non-neoplastic-novel cells, “ns” indicates that the enrichment fraction of Astrocyte is not significant compared with neoplastic-novel, and the P value is significant: *p<0.005, **p<0.0005. (C-F) t-SNE plots showing the expression of key genes in single-cell RNA sequencing data: (C) TCONS-00562265, (D) CD9, (E) CD44 and (F)

SOX2.

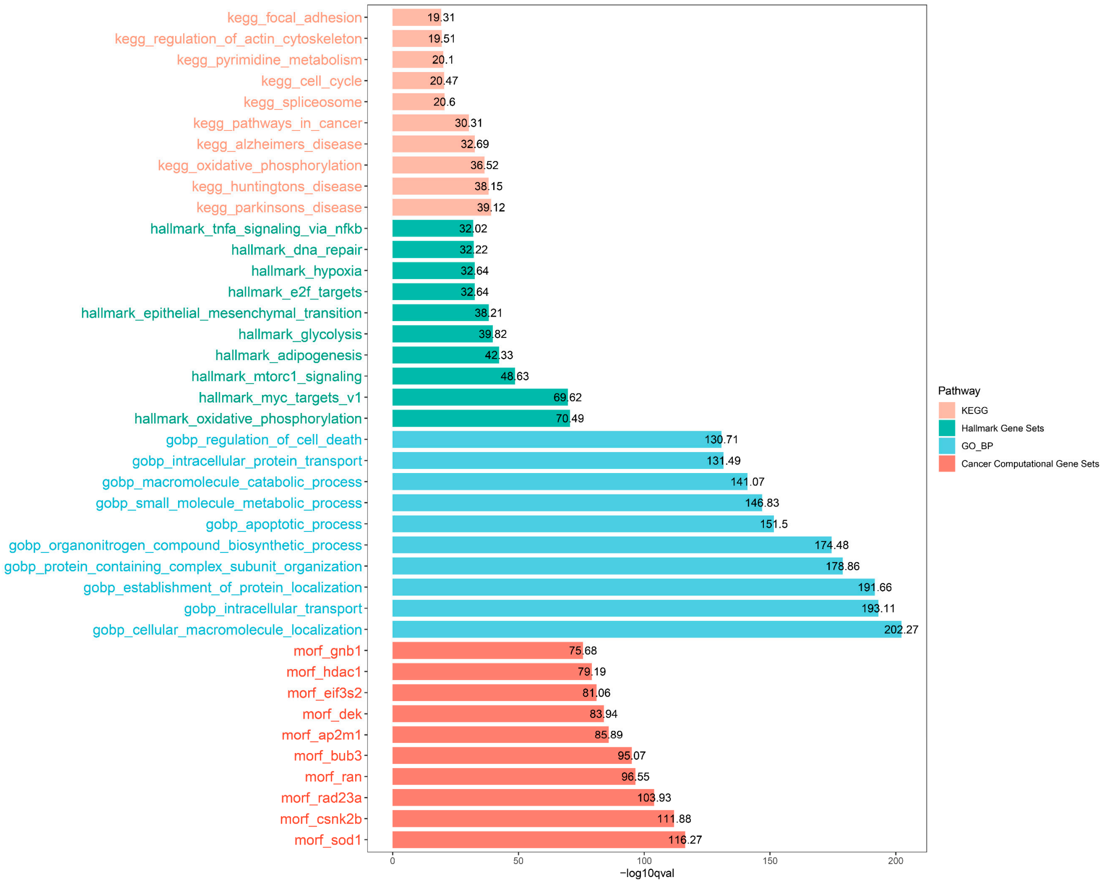

Supplementary Figure S2 | Enrichment analysis results showing that protein-coding genes co-expressed with TCONS-00562265 are enriched in the EMT pathway.

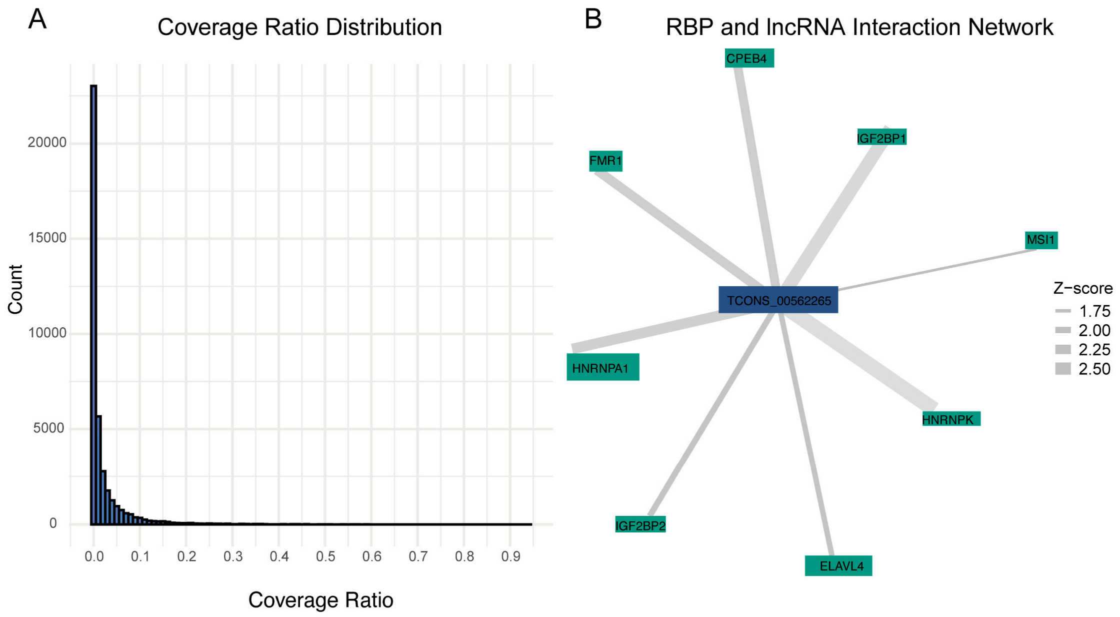

Supplementary Figure S3 | (A) Coverage ratio distribution histogram. Shows the proportion of riboseq data covering the unannotated lncRNA region. The horizontal axis is the coverage ratio, and

the vertical axis is the number of coverage ratios. (B) RBP and lncRNA Interaction Network. RBPs with significant binding sites to transcript TCONS-00562265, where the thickness of the edge represents the size of the Z-score.

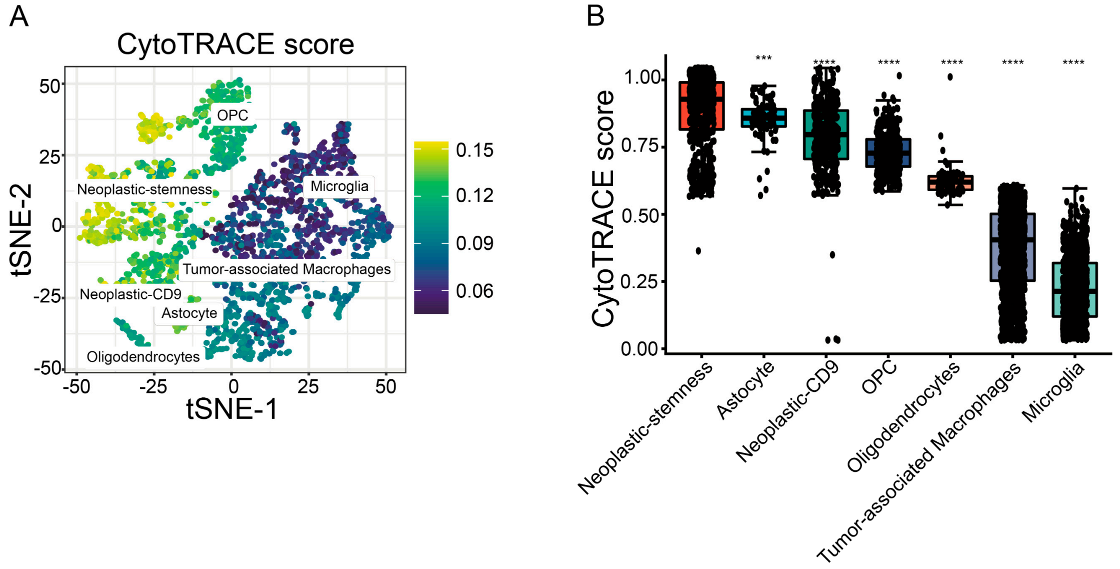

Supplementary Figure S4 | (A) t-SNE plot displaying the CytoTRACE cell differentiation potential scores. (B) Boxplot showing CytoTRACE scores across cell types, with higher scores (closer to 1) indicating greater differentiation potential. Asterisks denote significant differences in enrichment scores between neoplastic-novel and non-neoplastic-novel cells, while "ns" indicates no significant difference for Astrocytes compared to neoplastic-novel cells. Significance levels: **p*<0.005, ***p*<0.0005.

A

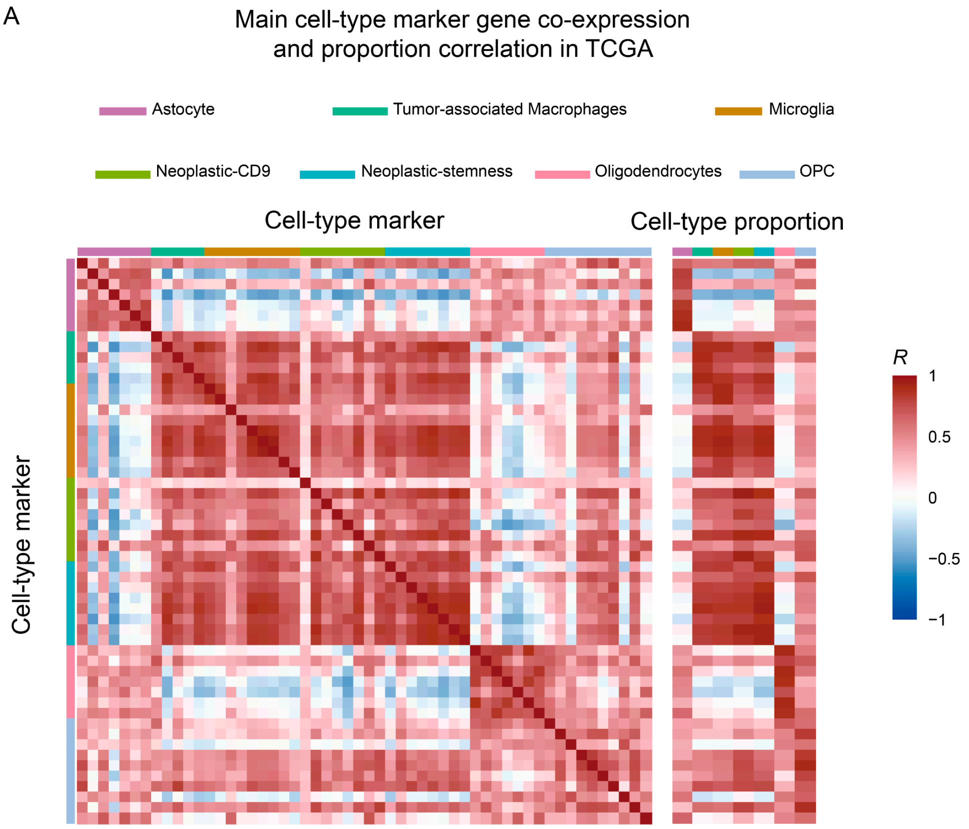

B

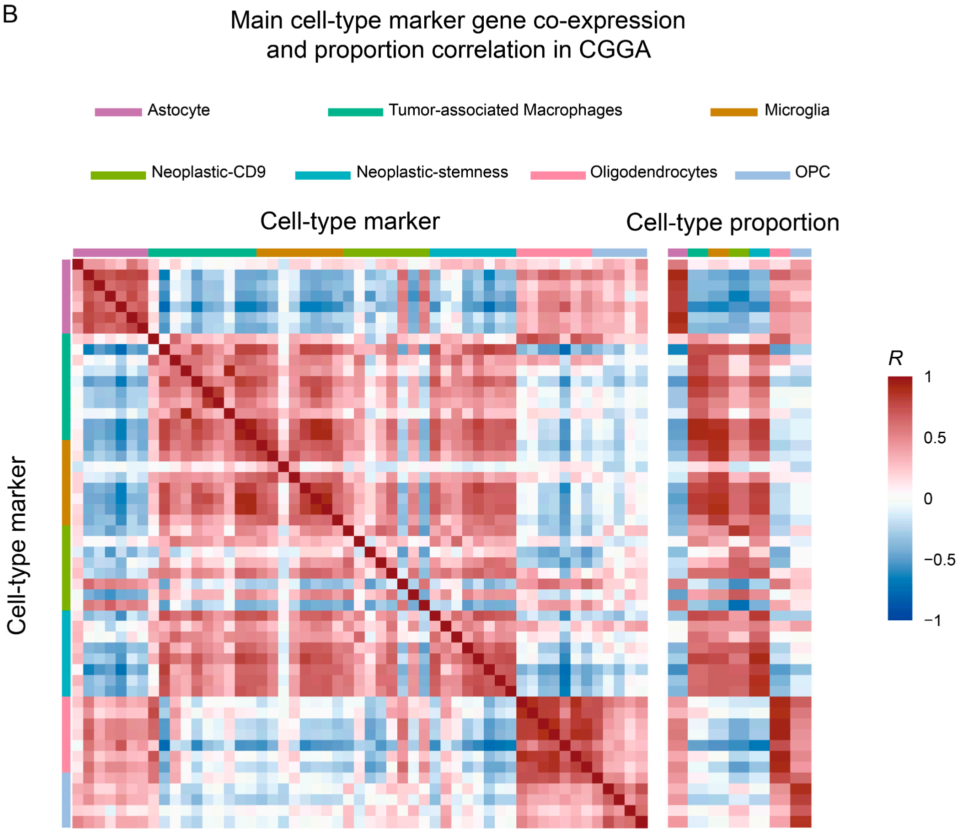

Supplementary Figure S5 | Correlation analysis of main cell-type marker gene co-expression and cell-type proportion. (A) In the TCGA dataset, marker genes for different cell types show strong intra-type co-expression and are highly correlated with their respective cell-type proportions,

indicating that these marker genes can effectively evaluate cell-type proportions. (B) Similar correlation patterns are observed in the CGGA dataset, further validating the robustness of marker genes in assessing cell-type proportions.

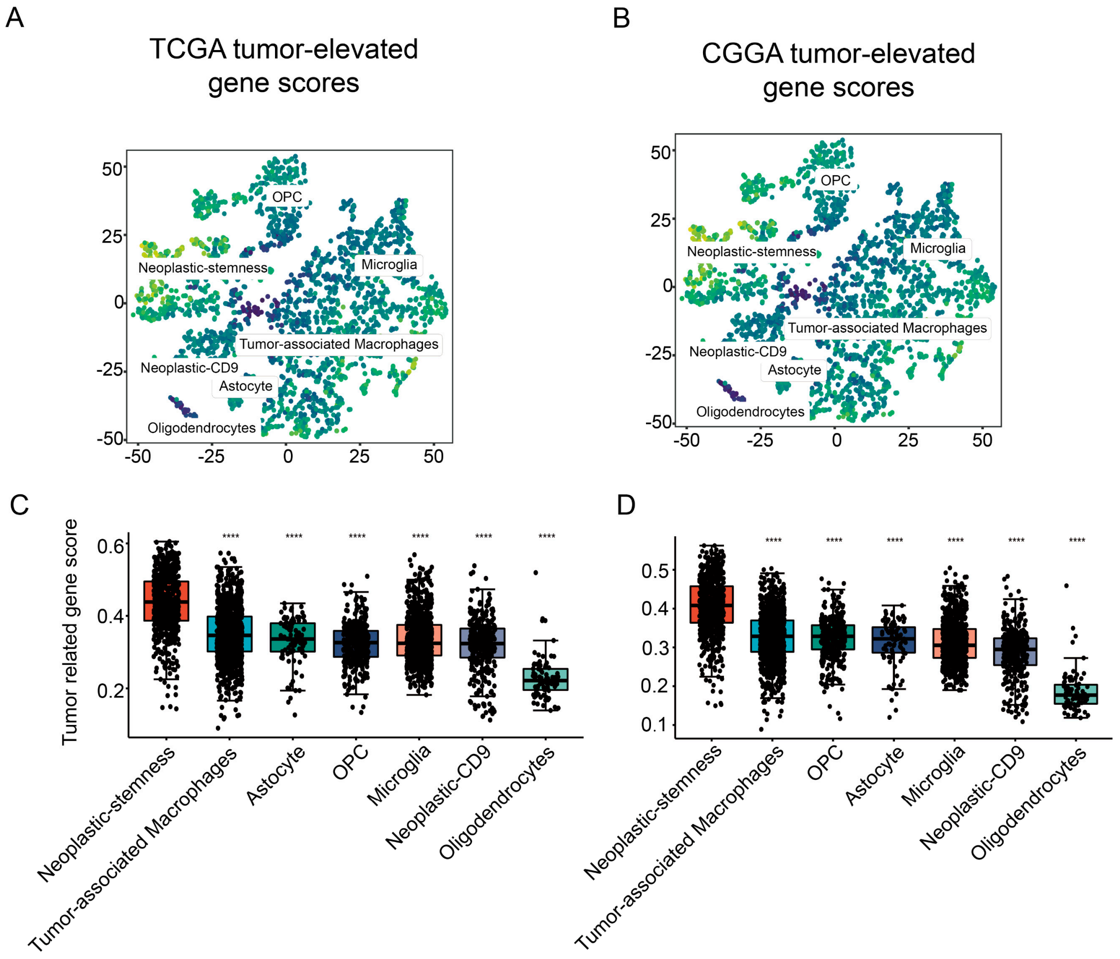

Supplementary Figure S6 | (A) and (B) UMAP plots from the TCGA and CGGA datasets show the AUC scores of tumor-elevated marker genes identified in single-cell GBM data. (C) and (D) Boxplots illustrate the AUC scores of tumor-elevated marker genes across various cell types in the TCGA and CGGA datasets. Tumor-elevated marker genes were identified using FindMarkers with FDR-adjusted p-values < 0.05, avg_log2FC > 0.5, and significant upregulation in bulk tumor samples. Asterisks indicate significant differences between neoplastic-stemness and non-neoplastic-stemness cells. Significance levels: *p<0.005, **p<0.0005.

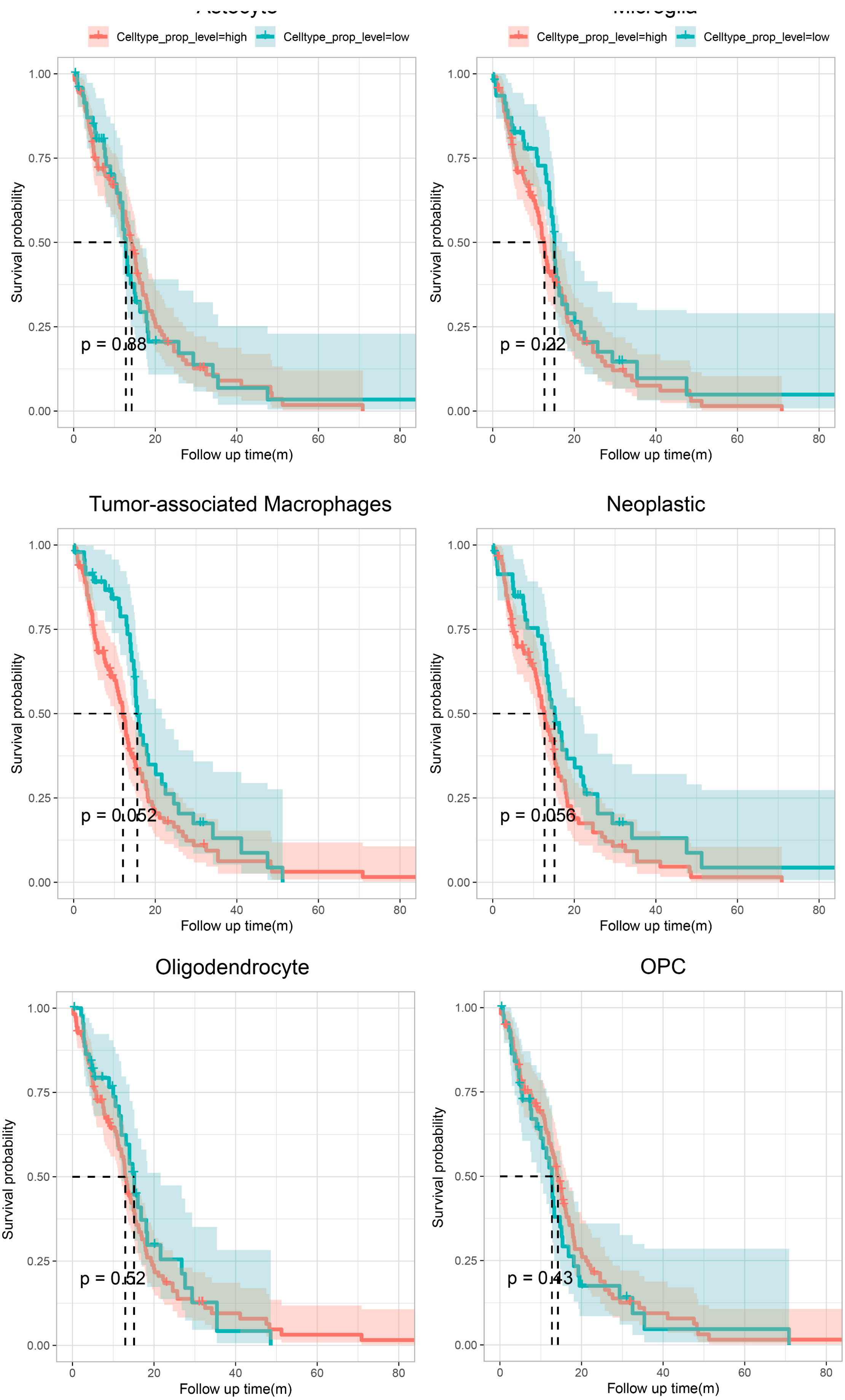

Celltype_prop_level=high
Celltype_prop_level=low
Survival probability
Follow up time(m)
p = 0.88
p = 0.22
Tumor-associated Macrophages
p = 0.052
Neoplastic
p = 0.056
Oligodendrocyte
p = 0.52
OPC
p = 0.43

Supplementary Figure S7 | Cox proportional hazards survival curves based on the proportion of different cell types, including Astrocytes, Microglia, Tumor-associated Macrophages, neoplastic cells, Oligodendrocytes, and OPCs in the TCGA dataset. High and low cell-type proportions are compared for each group. The p-values indicate no significant relationship between neoplastic cells and patient survival.

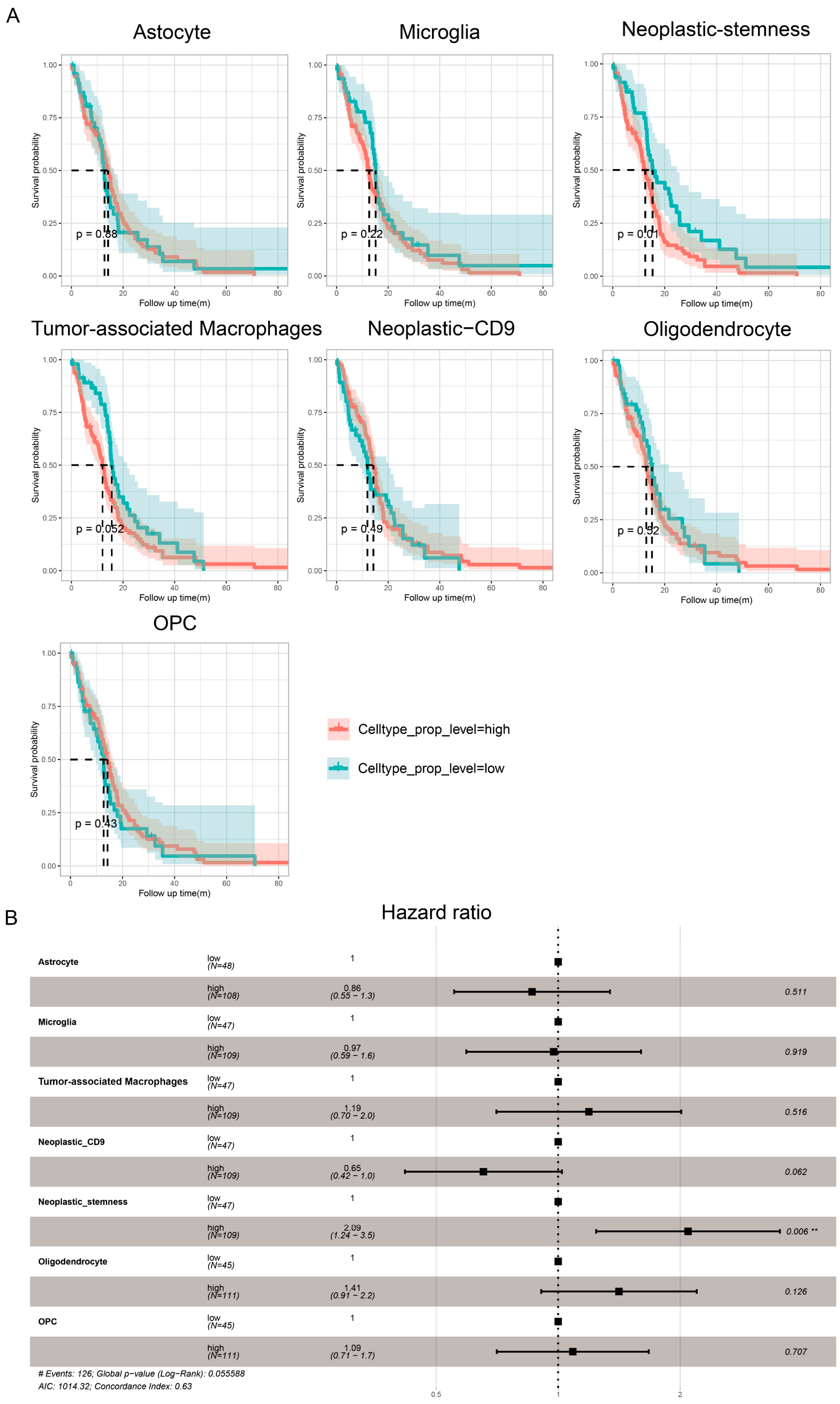
A
Astocyte
Microglia
Neoplastic-stemness
Tumor-associated Macrophages
Neoplastic−CD9
Oligodendrocyte
OPC
Survival probability
Follow up time(m)
p = 0.88
p = 0.22
p = 0.01
p = 0.052
p = 0.49
p = 0.52
p = 0.43
Celltype_prop_level=high
Celltype_prop_level=low
B
Hazard ratio
Astrocyte low (N=48) 1
high (N=108) 0.86 (0.55 − 1.3) 0.511
Microglia low (N=47) 1
high (N=109) 0.97 (0.59 − 1.6) 0.919
Tumor-associated Macrophages low (N=47) 1
high (N=109) 1.19 (0.70 − 2.0) 0.516
Neoplastic_CD9 low (N=47) 1
high (N=109) 0.65 (0.42 − 1.0) 0.062
Neoplastic_stemness low (N=47) 1
high (N=109) 2.09 (1.24 − 3.5) 0.006 **
Oligodendrocyte low (N=45) 1
high (N=111) 1.41 (0.91 − 2.2) 0.126
OPC low (N=45) 1
high (N=111) 1.09 (0.71 − 1.7) 0.707
Events: 126; Global p−value (Log−Rank): 0.055588
AIC: 1014.32; Concordance Index: 0.63
0.5
1
2

Supplementary Figure S8 | (A) Cox proportional hazards survival curves from the TCGA dataset showing the overall survival of patients stratified by high and low proportions of different cell types, including neoplastic-stemness and neoplastic-CD9 subtypes. Notably, patients with a high proportion of neoplastic-stemness cells exhibited significantly reduced overall survival ($p = 0.01$). In contrast, the proportion of neoplastic-CD9 cells showed no significant association with overall survival, similar to other cell types. (B) Multivariate hazard ratio analysis demonstrates that only a high proportion of neoplastic-stemness cells was significantly associated with an increased hazard ratio ($HR = 2.09$, $p = 0.006$), while the proportions of other cell types showed no significant impact on patient survival.

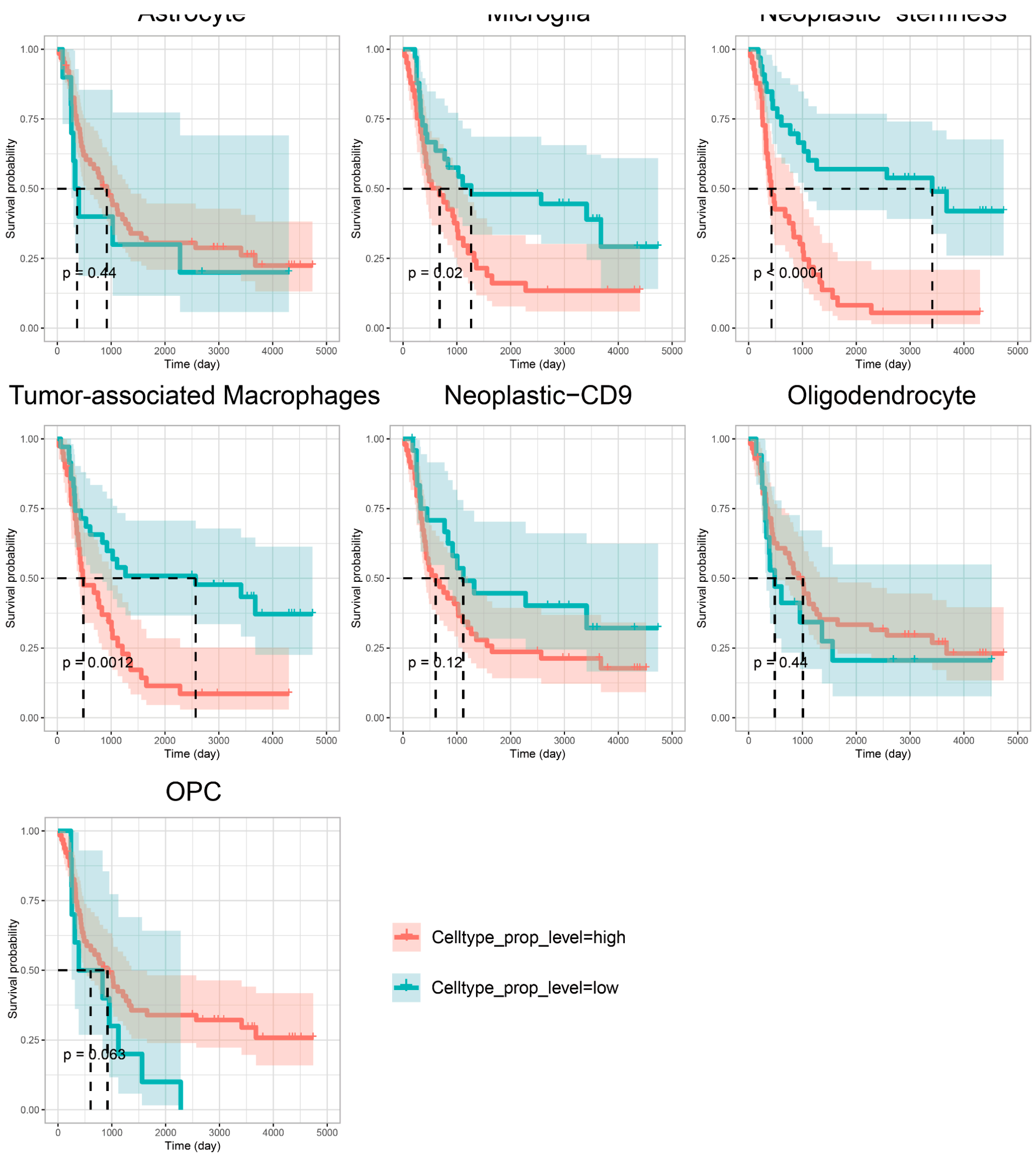

p = 0.44
p = 0.02
p < 0.0001
Tumor-associated Macrophages
p = 0.0012
Neoplastic−CD9
p = 0.12
Oligodendrocyte
p = 0.44
OPC
p = 0.063
Survival probability
Time (day)
Celltype_prop_level=high
Celltype_prop_level=low

B

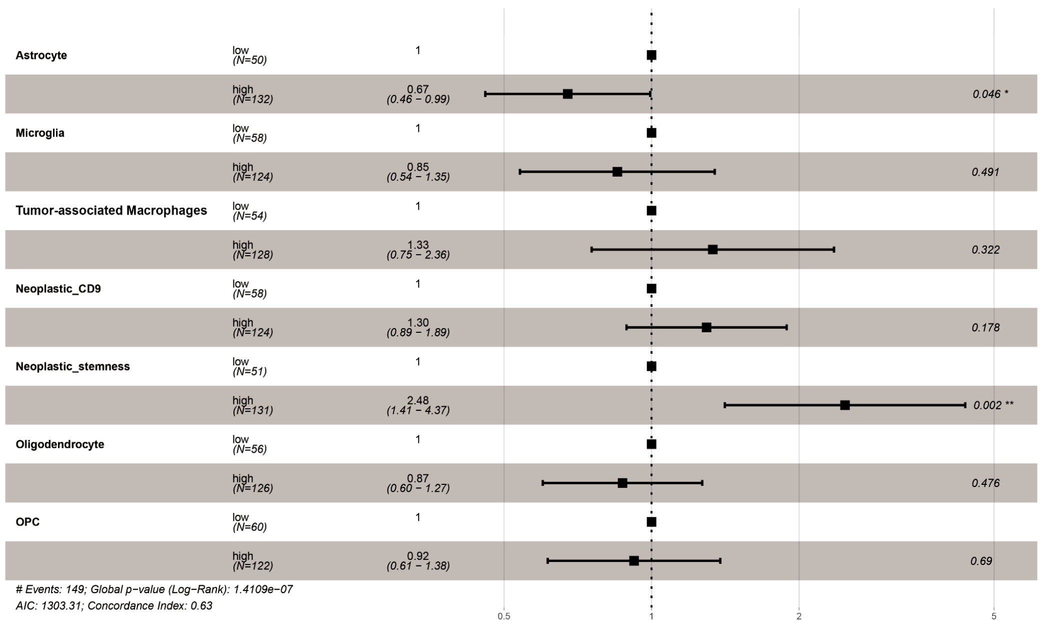

Hazard ratio
Astrocyte low (N=50) 1
high (N=132) 0.67 (0.46 − 0.99) 0.046 *
Microglia low (N=58) 1
high (N=124) 0.85 (0.54 − 1.35) 0.491
Tumor-associated Macrophages low (N=54) 1
high (N=128) 1.33 (0.75 − 2.36) 0.322
Neoplastic_CD9 low (N=58) 1
high (N=124) 1.30 (0.89 − 1.89) 0.178
Neoplastic_stemness low (N=51) 1
high (N=131) 2.48 (1.41 − 4.37) 0.002 **
Oligodendrocyte low (N=56) 1
high (N=126) 0.87 (0.60 − 1.27) 0.476
OPC low (N=60) 1
high (N=122) 0.92 (0.61 − 1.38) 0.69
Events: 149; Global p−value (Log−Rank): 1.4109e−07
AIC: 1303.31; Concordance Index: 0.63
0.5
1
2
5

Supplementary Figure S9 | (A) Cox proportional hazards survival curves of WHO Grade III glioma patients from the CGGA dataset, stratified by high and low proportions of various cell types, including neoplastic-stemness and neoplastic-CD9 subtypes. A significantly reduced overall survival was observed in patients with a high proportion of neoplastic-stemness cells ($p = 0.0001$), whereas no significant correlation was found between neoplastic-CD9 cells and survival ($p = 0.12$). Tumor-associated Macrophages also showed a significant impact on survival ($p = 0.0012$). (B) Multivariate hazard ratio analysis shows that only a high proportion of neoplastic-stemness cells is significantly associated with an increased hazard ratio (HR = 2.48, $p = 0.002$), while Astrocytes have a protective effect (HR = 0.67, $p = 0.046$).

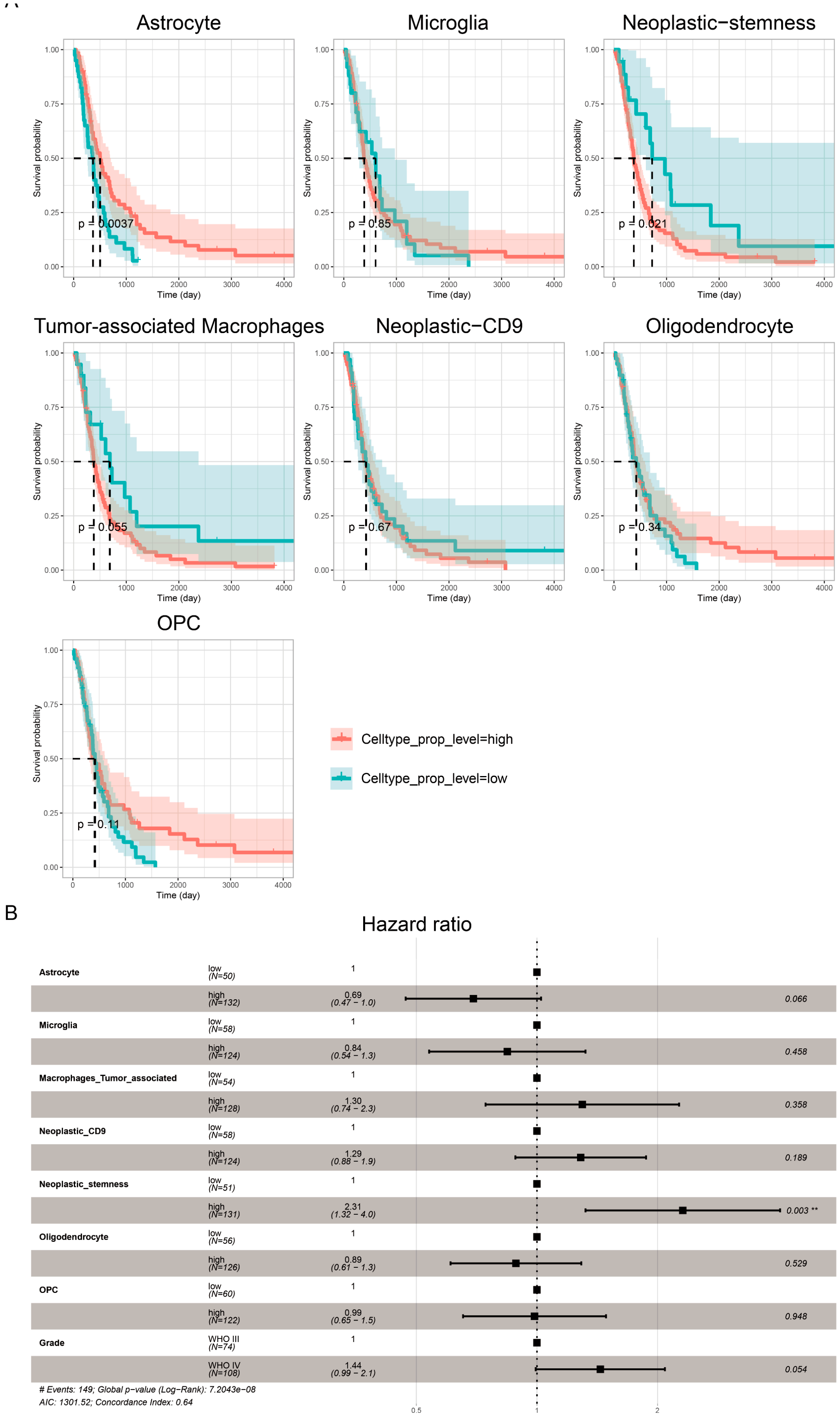
A
Astrocyte
Microglia
Neoplastic−stemness
Tumor-associated Macrophages
Neoplastic−CD9
Oligodendrocyte
OPC
Survival probability
Time (day)
p = 0.0037
p = 0.85
p = 0.021
p = 0.055
p = 0.67
p = 0.34
p = 0.11
Celltype_prop_level=high
Celltype_prop_level=low
B
Hazard ratio
Astrocyte
low (N=50) 1
high (N=132) 0.69 (0.47 − 1.0) 0.066
Microglia
low (N=58) 1
high (N=124) 0.84 (0.54 − 1.3) 0.458
Macrophages_Tumor_associated
low (N=54) 1
high (N=128) 1.30 (0.74 − 2.3) 0.358
Neoplastic_CD9
low (N=58) 1
high (N=124) 1.29 (0.88 − 1.9) 0.189
Neoplastic_stemness
low (N=51) 1
high (N=131) 2.31 (1.32 − 4.0) 0.003 **
Oligodendrocyte
low (N=56) 1
high (N=126) 0.89 (0.61 − 1.3) 0.529
OPC
low (N=60) 1
high (N=122) 0.99 (0.65 − 1.5) 0.948
Grade
WHO III (N=74) 1
WHO IV (N=108) 1.44 (0.99 − 2.1) 0.054
Events: 149; Global p-value (Log-Rank): 7.2043e−08
AIC: 1301.52; Concordance Index: 0.64

Supplementary Figure S10 | (A) Cox proportional hazards survival curves of WHO Grade IV glioma patients from the CGGA dataset, stratified by high and low proportions of various cell types. Patients with a high proportion of neoplastic-stemness cells exhibited significantly reduced overall survival (p = 0.021). Tumor-associated Macrophages show a trend toward significance (p = 0.055), while the proportions of other cell types, including neoplastic-CD9, did not significantly affect survival. (B) Multivariate hazard ratio analysis shows that a high proportion of neoplastic-stemness cells is significantly associated with an increased hazard ratio (HR = 2.31, p = 0.003), while other cell types do not show significant associations with survival outcomes.

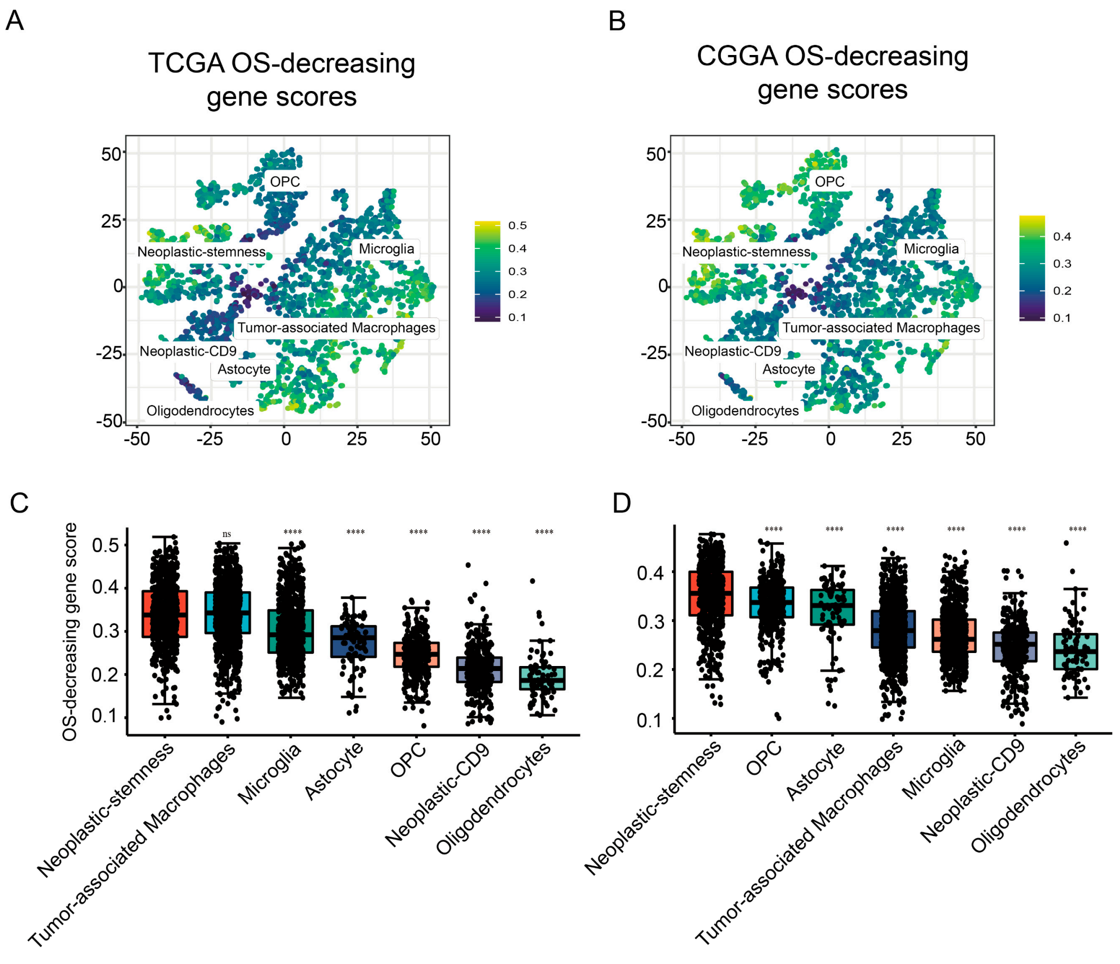

Supplementary Figure S11 | (A) and (B) t-SNE plots showing the enrichment scores of overall survival (OS)-decreasing genes in single-cell data from TCGA (A) and CGGA (B) patients. Higher enrichment scores are indicated by yellow coloring.(C) and (D) Boxplots comparing the enrichment scores of OS-decreasing genes between neoplastic-stemness and other cell types in TCGA (C) and CGGA (D) patients. Asterisks indicate a significant difference in gene scores between neoplastic-stemness and non-neoplastic-stemness cells, while "ns" denotes no significant difference.

Significance levels: *p < 0.005, **p < 0.0005.

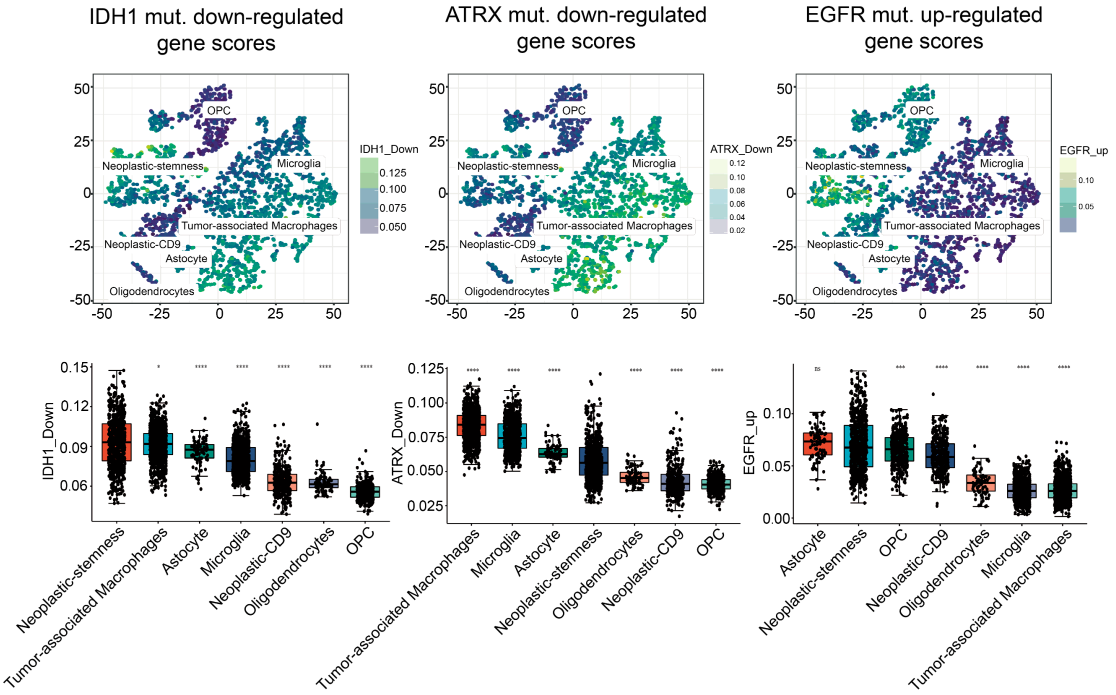

Supplementary Figure S12 | t-SNE plots showing the expression scores of down-regulated genes in IDH1-mutant (left) and ATRX-mutant (middle) samples, and up-regulated genes in EGFR-mutant (right) samples within single-cell data. Boxplots display the enrichment scores of these genes in different cell types. Asterisks denote significant differences in gene scores between neoplastic-stemness and other cell types, while "ns" indicates no significant difference. Significance levels: *p < 0.005, **p < 0.0005.

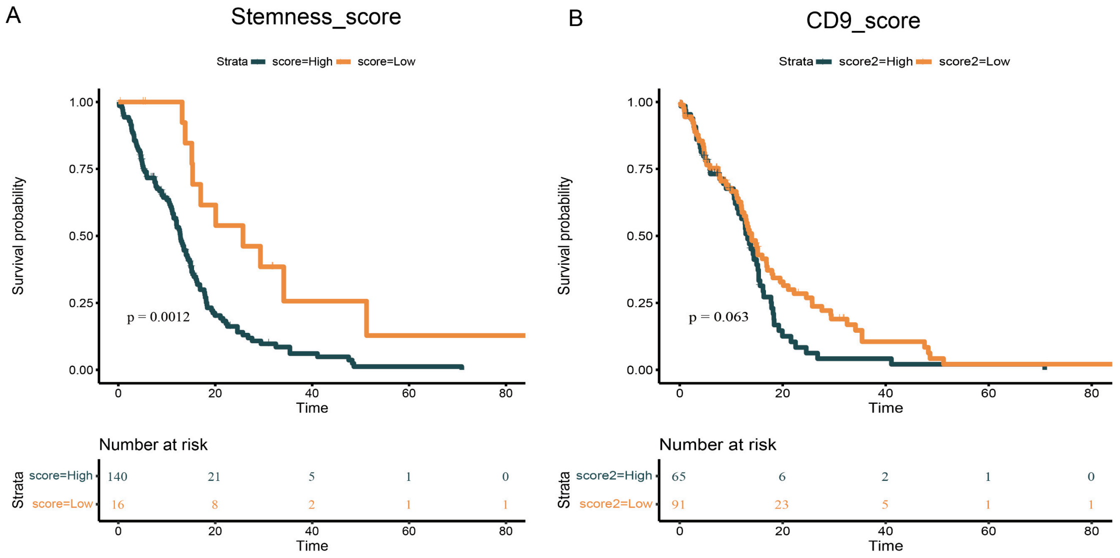

Supplementary Figure S13 | Cox proportional hazards survival curves based on (A) Stemness_score and (B) CD9_score. The Stemness_score was calculated using the marker genes of

neoplastic-stemness cells, applying PCA and extracting the first principal component as the feature score. Patients with a higher Stemness_score exhibited significantly worse overall survival ($p = 0.0012$), whereas the CD9_score showed no significant association with survival ($p = 0.063$).

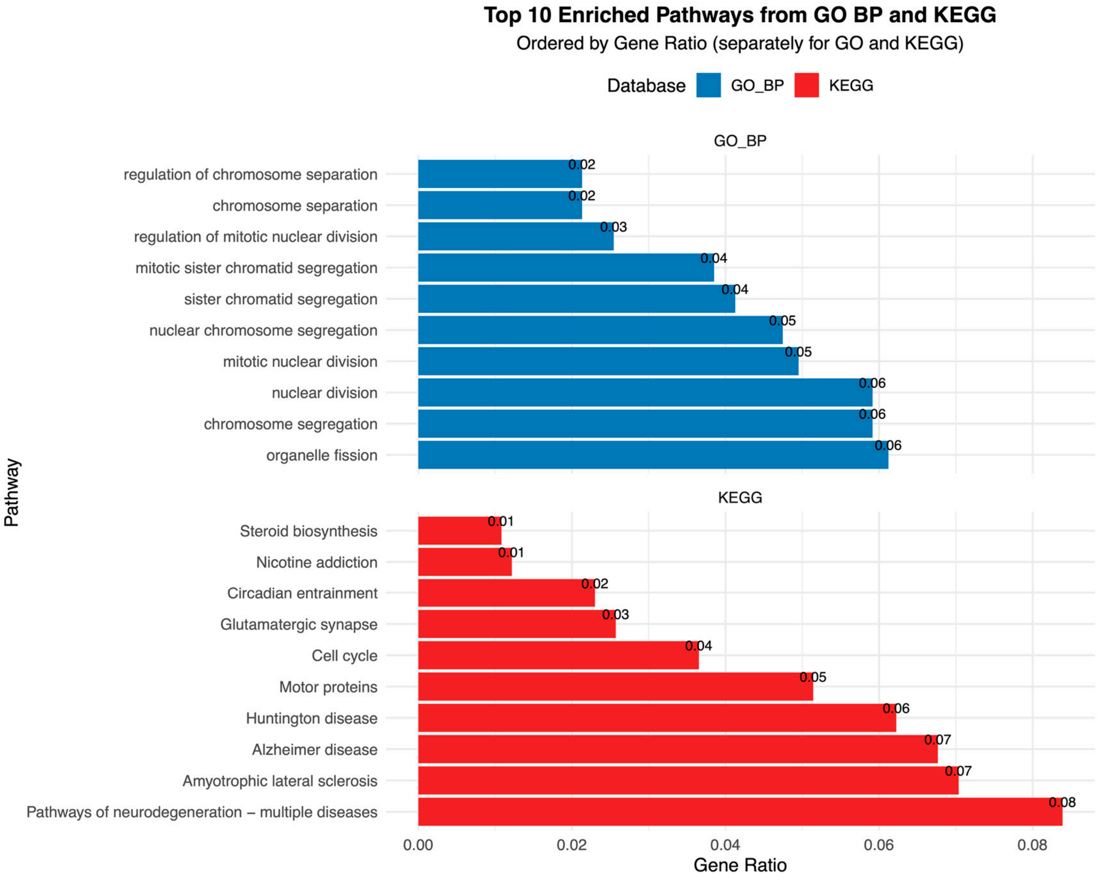

Supplementary Figure S14 | Functional enrichment analysis of downregulated lncRNAs in the hypoxic group. Enriched pathways are involved in cell migration, chromosome separation, nuclear division, and cell cycle regulation, indicating a reduction in proliferative capacity under hypoxic stress.

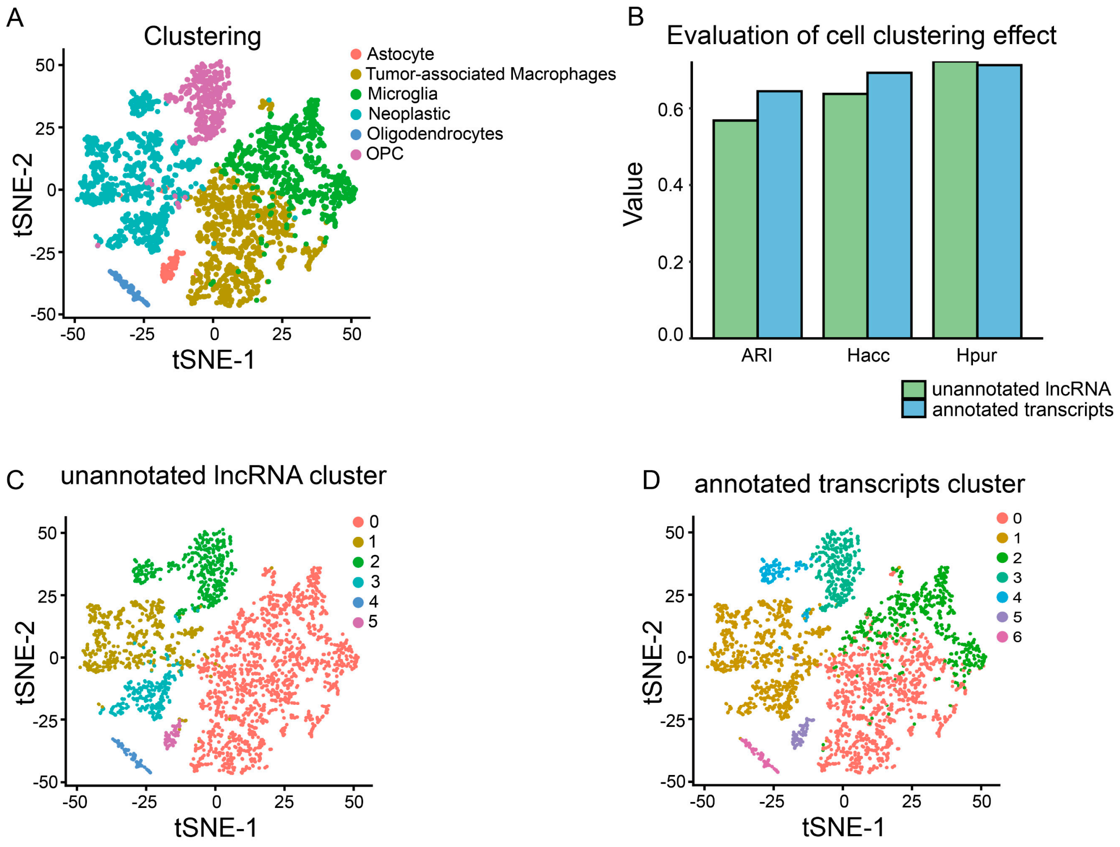

Supplementary Figure S15 | (A) t-SNE plot showing cell type annotations using all transcripts, both annotated and unannotated. (B) Evaluation of cell clustering performance using Adjusted Rand Index (ARI), clustering entropy (Hacc), and clustering purity (Hpur). Green bars represent unannotated lncRNAs, and blue bars represent annotated transcripts. Higher values indicate better clustering performance. (C) t-SNE plot showing cell clustering results using only unannotated lncRNAs. (D) t-SNE plot showing cell clustering results using only annotated transcripts.
